# Colorectal cancer mortality in persons with severe mental illness: a scoping review with meta-analyses of observational studies

**DOI:** 10.2340/1651-226X.2025.42260

**Published:** 2025-03-05

**Authors:** Paula R. Pop, Gitte S. Larsen, Mette K. Thomsen, Christoffer Johansen, Robert Zachariae, Bolette S. Rafn

**Affiliations:** a Danish Cancer Society National Research Center for Cancer Survivorship and Treatment Late Effects (CASTLE), Department of Oncology, Copenhagen University Hospital Rigshospitalet, Copenhagen, Denmark; b Department of Clinical Epidemiology, Aarhus University and Aarhus University Hospital, Aarhus, Denmark; c Danish Breast Cancer Group Center and Clinic for Late Effects (DCCL), Aarhus University Hospital, Aarhus, Denmark; d Unit for Psycho-oncology and Health Psychology, Department of Oncology, Aarhus University Hospital, Aarhus, Denmark; e Department of Psychology and Behavioural Sciences, Aarhus University, Aarhus, Denmark

**Keywords:** Colon cancer, psychosis, schizophrenia, affective disorders, health inequity, prognosis, survival

## Abstract

**Background and purpose:**

Persons with severe mental illnesses (SMIs) have reduced participation in colorectal cancer (CRC) screening programs, higher odds of advanced stage at diagnosis, and are less likely to receive adequate treatment than the general population. It remains unclear to what extent these factors impact CRC outcomes for persons with SMI. The aim of this scoping review was to describe and quantify CRC mortality for persons with SMI compared with the general population.

**Patients/materials and methods:**

We followed the *JBI Manual for Evidence Synthesis* and *PRISMA* guidelines in a systematic search of four databases from inception until April 29th, 2024. We included studies that provided CRC mortality estimates for adults with preexisting clinical diagnosis of SMI. We synthesized the results descriptively and pooled the data to estimate the magnitude of the associations.

**Results:**

Twenty-four original studies were identified with a total of 16.4 million persons. Most studies reported increased CRC mortality for persons with SMI compared with persons without SMI. The meta-analysis demonstrated a 25% increased CRC mortality for persons with SMI (e.g. pooled hazard ratio 1.25; 95% confidence interval 1.13 to 1.39; *n* = 13,178,161).

**Interpretation:**

The evidence points consistently to an increased CRC mortality for persons with SMI compared with persons without SMI. Furthermore, this evidence supports the idea that persons with SMI are a heterogenous population, and as such, any future initiatives to improve CRC outcomes for persons with SMI would warrant a tailored approach to potentiate individual resources, to mitigate stigma and structural discrimination.

## Introduction

Colorectal cancer (CRC) is the third most common cancer worldwide predominantly affecting adults 50 years and older [1]. Global trends for CRC have shown a decrease in both incidence and mortality [2]. Nevertheless, CRC is the second deadliest form of cancer after lung cancer. Similarly, CRC burden is second to lung cancer in its impact on disability-adjusted life-years in both middle-high and high sociodemographic countries [3, 4].

In the European Union (EU), population-based cancer screening programs are implemented for the early identification of breast cancer, cervical cancer, and CRC [5]. As of 2015, population-based CRC screening programs have been piloted or rolled out in 20 out of 28 EU members states [5]. However, uptake of cancer screening programs varies between population groups and is particularly low among those with mental illness [6, 7].

In 2019, the World Health Organization estimated that the prevalence of mental illness worldwide was 13% [8]. Meanwhile, the prevalence was 17% in the EU [9, 10]. ‘Severe or serious mental illness’ (SMI) is a subcategory of ‘any mental illness’ (AMI). SMI is distinguished from AMI in that the severity of the underlying mental illness results in a ‘functional impairment that substantially interferes with or limits one or more major life activities’, for example, basic daily living skills, instrumental living skills, and functioning in social, family, and vocational/educational contexts [11–14]. Mental illnesses that are frequently associated with functional impairment include schizophrenia, psychosis, major depression, and bipolar disorder [15]. As of 2021, the National Institute of Health reported the prevalence of SMI at 6% among US adults [16], an increase from earlier estimates of 4% [17].

Previous reviews have documented that persons with SMI have reduced participation in national CRC screening programs [6, 7], have higher odds of advanced disease stage at diagnosis [18], and experience inequity in the provision of adequate treatment (e.g. radiotherapy, chemotherapy, and sphincter-sparing surgery) [19].

While reported cancer incidences are equal to or in some cases lower for persons with SMI compared with the general population, cancer mortality, for many forms of cancer, is disproportionately higher (hazard ratio [HR]: 2.27 [2.15 to 2.39]) [20]. No previous reviews have summarized and synthesized data from single, original observational studies on the topic of CRC mortality in person with SMI compared with persons without SMI [19]. To address this gap in the review literature, we conducted this scoping review to describe CRC mortality for persons with SMI.

## Methods

We followed the methodological guidelines for conducting a scoping review specified in the *JBI Manual for Evidence Synthesis* [21] and the *Preferred Reporting Items for Systematic reviews and Meta-Analyses Extension for Scoping Reviews* [22] (Supplementary file 1).

An *a priori* protocol was registered with Open Science Framework Registries (osf.io/qv9kt).

### Eligibility criteria

#### Participants

We defined persons with SMI based on the Substance Abuse and Mental Health Services Administration (SAMHSA) definition [14]. We included adults aged ≥18 years, either with an established SMI diagnosis meeting International Statistical Classification of Diseases and Related Health Problems, 10th Revision (ICD-10) or Diagnostic and Statistical Manual of Mental Disorders, 4th edition (DSM-IV) diagnostic criteria or currently receiving treatment for SMI. We selected the mental disorders placed higher in the ICD-10 classification, and we cross-referenced these disorders with adult hospitalization reports and disability entitlement status reports. We selected the following mental disorder categories (ICD-10) as representative for SMI: schizophrenia, including psychosis (F20-29), mood [affective] disorders (F30-39), anxiety disorders, including post-traumatic stress disorder (PTSD) (F40-48), eating disorders (F50), and specific personality disorders (F60). Finally, SMI status had to precede the CRC diagnosis.

We excluded persons with functional impairment resulting from mental disorders that were developmental, neurological, or substance use disorders and cases where mental illness was assessed using screening questionnaires, for example, Hospital Anxiety and Depression Scale (Supplementary file 2).

#### Concept

Disease prognosis can be expressed in terms of metastasis, local recurrence, mortality, or survival. We used the two latter characteristics and defined CRC mortality as the risk of dying from a primary cancer localized to the colon, rectum, rectosigmoid, and anus regions. Similarly, CRC survival was defined as the probability of being alive following a primary diagnosis and treatment of cancer with the same topography. To be included, studies had to report CRC-specific mortality and/or survival estimates. Thus, we excluded all studies reporting only all-cause mortality and overall survival estimates in the SMI populations under study.

#### Context

No exclusions were made based on the context.

### Search

Four databases, namely, Cumulative Index to Nursing and Allied Health Literature (CINAHL), Excerpta Medica Database (EMBASE), MEDLINE, and Web of Science, were searched from inception until April 29th, 2024. Google Scholar was also searched. Finally, citation searches were performed of all included articles following full text screening.

The search strategy was developed by translating the research question into PICO elements (i.e., patient, intervention, comparator, and outcome) elements, followed by the identification of specific concepts as free text elements and database-specific headings and subheadings. Multiple searches were performed in each database for both broader and narrower concepts. The research team reviewed all search strategies, and the final strategy was selected by consensus (Supplementary files 3 and 4) [23].

### Selection of sources of evidence

All search results were uploaded to *Covidence* [24]. Two reviewers independently screened the title/abstract and full text following preestablished eligibility criteria. Group discussion was used to resolve conflicts.

### Data charting process

The data extraction template was based on *JBI Manual for Evidence Synthesis* [25] and Effective Practice and Organisation of Care (EPOC) [26] resources (Supplementary file 5). Two reviewers extracted data, that is, the first reviewer extracted data; the second reviewer verified the extracted data. The corresponding authors were contacted to request additional information, which was used to determine eligibility of the article and for data synthesis.

### Critical appraisal

A formal quality assessment of the included studies is generally not performed in scoping reviews [25]. We therefore had to rely on the rigor of the review process especially having to focus on fulfillment of all inclusion and exclusion criteria. We screened all included articles for the statement of adherence to formal established reporting guidelines (yes/no), for example, *STrengthening the Reporting of OBservational studies in Epidemiology* (STROBE) [27].

### Synthesis of results

Given the high population heterogeneity and relatively small sample sizes of persons with SMI and CRC-deaths, the results were summarized narratively and in a table form by the study type (cohort, register study, single center, and population-based), SMI category (multiple diagnostic groups and single diagnostic group), CRC type (all CRC, only colon, and only rectum), and types of effect estimates. Indirect data, meaning data not directly extracted from the studies, but calculated based on reported sample sizes (*n*) were used when needed.

Data for three reported estimate types, that is, HRs, standardized mortality ratios (SMRs), and risk ratios (RRs) calculated based on events at the population size, were subjected to meta-analysis to ascertain the pooled overall effect sizes (ESs) and their precision. Inverse variance-weighted random-effects models taking the precision of each study into consideration were used in all meta-analyses, with HRs, SMRs, and RRs larger than 1.0 taken to indicate an association of SMI in the direction of increased CRC mortality. Care was taken to ensure that the included results represented independent samples, and results reporting findings of the same or overlapping samples or subsamples were excluded from the analyses. The individual and pooled ESs are presented together with the associated 95% confidence intervals (CIs) in forest plots.

Heterogeneity, that is, the degree to which the variation in ESs reflects true differences (heterogeneity) or sampling error, was investigated using Q and *I*^2^ statistics [28, 29]. If the results indicated heterogeneity (*I*^2^ > 0.0), we calculated the 95% prediction interval, which estimates the expected range of true effects in 95% of future studies [30].

The possibility of publication bias was assessed using funnel plots and Egger’s test for pooled results of 10 or more ESs [31]. If results were suggestive of possible publication bias, we planned to conduct sensitivity analyses by imputing the ‘missing studies’ and calculating adjusted ESs using the Duval and Tweedie trim-and-fill method [32]. The analyses were performed using Comprehensive Meta-Analysis, version 4 [33].

## Results

**Figure 1:**
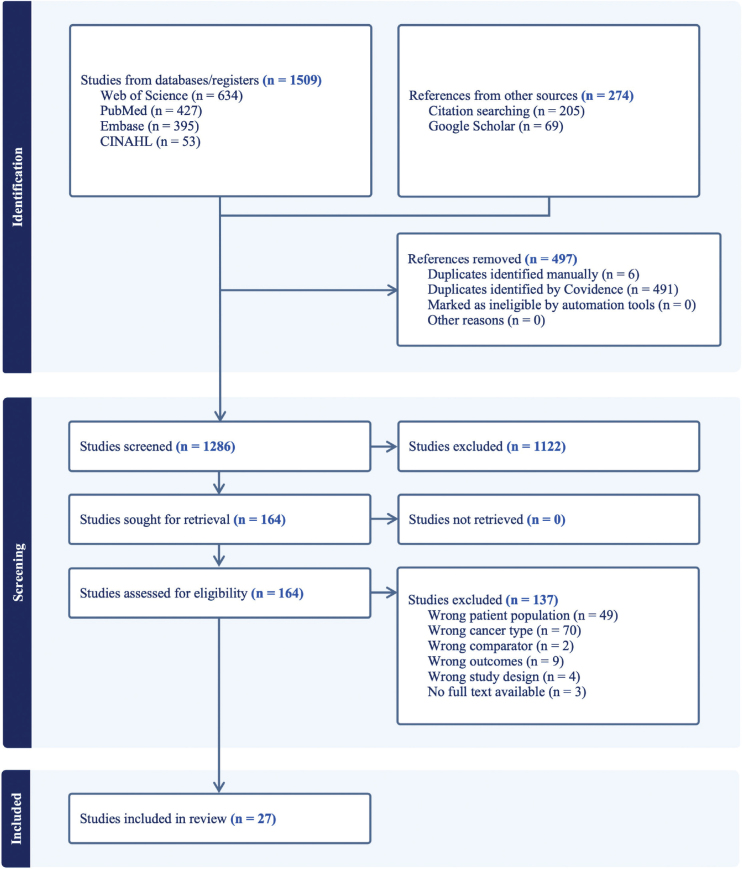
PRISMA flow diagram.

Of the 1783 identified articles, 27 articles reporting on 24 independent samples met all inclusion criteria (Figure 1). Information on excluded studies and reasons for exclusion is available as Supplementary file 6. Study characteristics are summarized according to the study design, population, cancer site, and effect estimate in Table 1.

**Table 1:** Study overview.^a^

Author, year, region	Design, follow-up period	No. of subjects	Comparisons N Exposed/N Control	Primary cancer site	Data sources
Katz et al., 1967, USA [34]	Retrospective cohort 1955–1961	3,365	Mental hospital population vs. general population 3,365/NA	Rectum	New York State Department of Mental Hygiene, Office of Vital Statistics of the New York State Department of Health
Saku et al., 1995, Japan [35]	Retrospective cohort Single center: National Mental Hospital, Saga 1948–1982	4,980	Persons with schizophrenia vs. general population 2,268/NA	Colon, rectum, rectosigmoid junction, and anus	Koseki Japanese family register
Dalton et al., Dalton et al., Egeberg et al., 2008, Denmark [36–38]	Retrospective cohort Population-based 1994–2006	3,218,440	Persons with psychiatric comorbidity (schizophrenia and other psychoses, depression) vs. persons without mental illness 555/16,814	Colon, rectum, rectosigmoid junction, and anus	Central Population Register, Danish Cancer Register, NORDCAN database, Integrated Database for Labour Market Research in Statistics Denmark, Register for Education Statistics, the Register Relating to Unemployment, Building and Dwelling Register, Danish National Patient Register, Psychiatric Case Register
Tran et al., 2009, France [59]	Prospective cohort 1993–2003	3,434	Persons with schizophrenia vs. general population 3,434/NA	Colon	French public departments of adult psychiatry (*n* = 122)
Baillargeon et al., 2011, USA [39]	Retrospective cohort 1993–2005	80,670	Persons (≥ 67 years) with mood disorders, psychotic disorders, and other mental disorders vs. persons without mental illness 20,699/59,971	Colon	Surveillance, Epidemiology and End Results (SEER)-Medicare, Social Security Administration
Crump et al., 2013a, Sweden [40]	Retrospective cohort Population-based 2001–2009	6,587,036	Persons with bipolar disorder vs. general population 6,618/ 6,587,036	Colon	Swedish Outpatient Registry, Swedish Hospital Registry, Swedish Pharmacy Registry
Crump et al., 2013b, Sweden [41]	Retrospective cohort Population-based 2001–2009	6,097,834	Persons with schizophrenia vs. general population 8,277/6,097,834	Colon	Swedish Outpatient Registry, Swedish Hospital Registry, Swedish Pharmacy Registry
Perini et al., 2014, Italy [42]	Retrospective cohort 1982–2006	9,931	Persons with schizophrenia, affective disorders, neurosis, personality disorders vs. regional population 9,931/NA	Colon and rectum	Central Person Registry of the Municipalities of Verona, Castel d’Azzano, and Buttapietra; Mortality Registry of the Local Health District of Verona, Registries of Deaths of the Municipalities of Verona, Castel d’Azzano, and Buttapietra and of the Service of Forensic Medicine of the University of Verona
Cunningham et al., 2015, New Zealand [43]	Retrospective cohort 2006–2010	4,022	Persons with recent mental illness (functional psychosis and contact with mental health services for other reasons) vs. persons without mental illness 190/3,822	Colorectal	New Zealand Ministry of Health database, National Health Index database, New Zealand Cancer Registry, Mental Health Information National Collection, Project for Integration of Mental Health Data, New Zealand Mortality Data Collection, National Minimum Data Set
Olfson et al., 2015, USA [44]	Retrospective cohort 2001–2007	1,138,853	Persons with schizophrenia vs. general population 1,138,853/NA	Colon	US Compressed Mortality File, United States Life Tables, Medicaid Analytic eXtract (MAX) data from the Centers for Medicare & Medicaid Services, National Death Index (NDI), Medicaid eligibility data
Sun et al., 2015, Denmark [45]	Retrospective cohort 2003–2010	201,662	Persons using antidepressant (AD) vs. persons not using AD 201,662/168,551	Colorectal (lower digestive organs)	Danish Cancer Registry, Danish National Prescription Registry, Danish Civil Register, Integrated Database for Labour Market Research, Danish Hospital Register
Manderbacka et al., 2018, Finland [46]	Retrospective cohort 1990–2013	40,799	Persons with SMI (schizophrenia, mood disorders) vs. persons without mental illness 1,473/ 39,326	Colorectal	Finnish Cancer Registry, Causes of Death statistics maintained by Statistics Finland, Hospital Discharge Register
Toender et al., 2018, Denmark [47]	Retrospective cohort Population-based 1978–2013	5,899,592	Persons with SMI (schizophrenia, schizoaffective disorder, or bipolar affective disorder) vs. general population 4,307/574,732	Colorectal	Danish Civil Registration System, the Danish Psychiatric Central Research Register, Danish Cancer Register
^a^Klaassen et al., 2019, Canada [48]	Retrospective cohort Population-based 1997–2014	676,125	Persons with a history of psychiatric utilization (PU) vs. persons without mental illness 316,660/359,465	Colorectal	Institute for Clinical Evaluative Sciences, Ontario Cancer Registry (OCR), Registered Persons database, Ontario Health Insurance Plan (OHIP)
Harris et al., 2020, USA [49]	Retrospective cohort 2004–2013	112,283	Older persons with mental illness (depression, anxiety, psychotic, or bipolar disorder) vs. persons without mental illness 23,726/88,557	Colorectal and anus	Surveillance, Epidemiology, and End Results (SEER)-Medicare linked database
Liang et al., 2020, USA [60]	Historic cohort 1993–2018	2,396	Postmenopausal women with depression (symptoms or AD use) vs. women without depression 2,396/NA	Colorectal	Women’s Health Initiative (WHI) Observational Study (OS) and Clinical Trial (CT)
^a^Mahar et al., 2020 & 2021, Canada [50, 51]	Retrospective cohort 2007–2012/2013	24,507	Persons with SMI (major depression, bipolar disorder, schizophrenia, or other nonorganic psychotic illnesses) vs. persons without mental illness 740/23,767	Colorectal	Ontario Cancer Registry (OCR), Canadian Institute for Health Information (CIHI), Discharge Abstract Database (DAD), Ontario Mental Health Reporting System (OMHRS), Ontario Health Insurance Plan (OHIP) database, ICES Physician Database, National Ambulatory Care Reporting System (NACRS), Cancer Care Ontario’s Activity Level Reporting database, Registered Persons Database, Ontario Registrar General database
Grassi et al., 2021, Italy [52]	Retrospective cohort, Population-based 2008–2017	12,385	Persons with severe mental disorders (schizophrenia or other functional psychosis, mania, or bipolar affective disorders) vs. regional population 12,385/NA	Colon, rectosigmoid junction, rectum, and anus	Emilia-Romagna Mental Health Registry, Regional Cause of Death Registry, Regional Population Archive
Launders et al., 2022, UK [53]	Retrospective cohort, Matched 1:4 2000–2018	348,160	Persons with schizophrenia, bipolar disorder or other non-organic psychoses vs. persons without mental illness 69,632/278,528	Bowel cancer	Clinical Practice Research Datalink (CPRD) GOLD and Aurum databases
Cheng et al., 2023, Taiwan [54]	Retrospective cohort Matched 1:4 2000–2019	537,415	Persons with schizophrenia vs. general population 107,481/429,924	Colon and rectum	The National Health Insurance Research Database, The Taiwan Cancer Registry
Chierzi et al., 2023, Italy [55]	Retrospective cohort, Population-based 2008–2017	101,487	Common mental disorders: depression or neurotic disorders vs. regional population 101,487/NA	Colon, rectosigmoid junction, rectum, and anus	Emilia-Romagna Mental Health Registry, Regional Cause of Death Registry, Regional Population Archive
^a^Oh et al., 2023, South Korea [56]	Retrospective cohort Population-based 2002–2019	944,794	Preoperative psychiatric morbidity: depression, anxiety disorder, and post-traumatic stress disorder vs. persons without mental illness 24,370/920,424	Colorectal	South Korean NHIS database
Drevinskaite et al., 2024, Lithuania [57]	Retrospective cohort Single center: Vilnius Republican Psychiatric Hospital 1992–2020	8,553	Persons with schizophrenia vs. national mortality rates 8,553/NA	Colon, rectum, rectosigmoid, and anus	Central Population Register, Lithuanian Cancer Register
^a^Wootten et al., 2024, Canada [58]	Retrospective cohort Population-based 1995–2019	24,942	Persons with non-affective psychotic disorders (schizophrenia, schizoaffective disorder, schizophreniform, or psychosis not otherwise specified) vs. persons without mental illness 5,002/19,940	Colorectal	Registered Persons Database (RPDB), Ontario Cancer Registry (OCR)

AD: antidepressant; NA: not available; PD: psychiatric disorder; PU: psychiatric utilization; SMI: severe mental illness.

a Studies including a reporting guidelines statement, STROBE guidelines or similar.

### Characteristics of sources of evidence

#### Study types

All 24 original studies were cohort studies, of which 22 applied administrative data linkage [34–58]. Among these cohorts, two were single center [35, 57] and eight were population-based [38, 40, 41, 47, 48, 52, 56, 58]. The studies were published between 1967 and 2024 and represented populations from Europe (*n* = 12) [38, 40–42, 45–47, 52, 53, 55, 57, 59], North America (*n* = 8) [34, 39, 44, 48–50, 58, 60], East Asia (*n* = 3) [35, 54, 56], and Oceania (*n* = 1) [43]. The average follow-up period was 16 years, with the longest follow-up of 36 years [47].

### Population

There were two main approaches in defining the population. Most studies identified a cohort of persons with SMI (*n* = 16) [34, 35, 40–44, 47, 52–59], which was linked to CRC-death. The remaining studies identified a cohort of persons with cancer (*n* = 8) [38, 39, 45, 46, 48–50, 60], that is, various forms of cancers, including CRC, which were linked both to SMI as exposure variable preceding cancer diagnosis and to CRC-death.

Nine studies selected a single SMI diagnosis population, for example, bipolar disorder (*n* = 1) [40], depression (*n* = 1) [60], and schizophrenia (*n* = 7) [35, 41, 44, 54, 57–59]. Additionally, one study [45] assessed the effect of exposure to antidepressants in the 3 years preceding cancer diagnosis on CRC mortality. The remaining studies sampled mixed SMI groups with diagnoses, such as anxiety disorder, bipolar disorder, depression, mania, neurotic disorders, functional and nonorganic psychosis, schizoaffective disorder, schizophrenia, and other psychiatric diagnoses. Several authors categorized SMI according to either severity or service utilization, for example, inpatient versus outpatient [50, 51], psychiatric service utilization gradient [48], SMI, or AMI [43].

### Cancer site

CRC diagnosis was reported as colon cancer; rectal cancer; CRC (colon cancer *and* rectum cancer); CRC *and* anal cancer; colon, rectosigmoid, rectum, *and* anus cancers. Six studies primarily reported on CRC [34, 36–38, 46, 50, 51, 60]. The remaining studies reported CRC mortality alongside other cancers [45, 48, 49].

### Effect estimates

CRC mortality was assessed using mortality rates (MRs), mortality rate ratios (MRRs), mortality-to-incidence ratio (MIR), and CRC-specific SMRs. Additionally, survival was assessed as 1- and 5-year relative survival percent of expected survival (RS), absolute survival (additional deaths per 1,000 person-years), and proportional hazard analysis model (HR). Table 2 presents an overview of the extracted data from each study included.

**Table 2:** Risk estimates extraction.

Author, year	Cancer site	Risk estimates		
Katz 1967 [34]	Rectum	**Observed deaths as % of expected deaths**		
		Total 90.2%	Men 83.3% Women 98.2%	< 65 years 129.3% > 65 years 72.4%
Saku 1995 [35]	Colon, rectum, rectosigmoid junction, anus	**SMR 95% CI**		
		**C18 (colon cancer)** Men 0.95 (0.02, 5.29) Women 3.58 (0.43, 12.9)	**C19-21 (rectosigmoid junction, rectum, and anus cancers)** Men 2.71 (0.56, 7.93) Women NA	
Dalton 2008a Dalton 2008b Egeberg 2008 [36–38]	Colon	**1-year/5-years relative survival % (95% CI) (observed survival as % of expected survival)**		
		Depression Men 67 (58–78)/ 48 (47–50) Women 77 (70–84)/ 50 (42–60) Controls for patients with depression Men 72 (71–73)/ 44 (43–46) Women 74 (73–74) 48 (47–50)	Schizophrenia Men 58 (42–79)/ 28 (15–51) Women 76 (65–89)/47 (35–65) Controls for patients with schizophrenia Men 72 (71–73)/ 44 (43–46) Women 74 (73–76)/ 48 (47–50)	
	Rectum	**1-year/5-years relative survival % (95% CI) (observed survival as % of expected survival)**		
		Depression Men 79 (70–90)/ 45 (34–59) Women 86 (79–94)/ 51 (40–65) Controls for patients with depression Men 79 (78–80)/ 46 (45–48) Women 82 (81–84)/ 53 (51–55)	Schizophrenia Men 51 (36–72)/ 27 (16–46) Women 60 (46–77)/ 34 (21–52) Controls for patients with schizophrenia Men 79 (78–80)/ 46 (45–48) Women 83 (81–84)/ 53 (51–55)	
Tran 2009 [59]	Colon	**SMR 95% CI**		
		Total 2.20 (0.80, 4.80) Men 0.70 (0.03, 3.29) Women 5.00 (2.10–12.0)		
Baillargeon 2011 [39]	Colon	**HR 95% CI**		
		**Model 1** Mood disorders 1.02 (0.97, 1.06) Psychotic disorders 1.27 (1.20, 1.36) Other mental disorders 1.01 (0.96, 1.05)	**Model 2** Mood disorders 1.03 (0.99, 1.08) Psychotic disorders 1.19 (1.11, 1.27) Other mental disorders 0.99 (0.95, 1.04)	**Model 3** Mood disorders 1.04 (0.99, 1.10) Psychotic disorders 1.16 (1.10, 1.24) Other mental disorders 1.03 (0.99, 1.08)
		Model 1: Unadjusted HR (95% CI); Model 2: Adjusted HR (95% CI) Model 1 adjusted for age; race and ethnicity; sex; marital status; Surveillance, Epidemiology and End Results region; income; and year of diagnosis; Model 3: Adjusted HR (95% CI) Model 2 additionally adjusted for comorbid disease index and cancer stage at diagnosis index.		
Crump 2013a [40]	Colon	**HR 95% CI**		
		**Model 1** Men 1.96 (0.98, 3.92) Women 2.16 (1.26, 3.73)	**Model 2** Men 1.82 (0.91, 3.65) Women 2.08 (1.21, 3.59)	**Model 3** Men 1.83 (0.92, 3.67) Women 2.09 (1.21, 3.6)
		Model 1: Adjusted hazard ratio (95% CI), age adjusted; Model 2: Adjusted hazard ratio (95% CI), age and other sociodemographic; Model 3: Adjusted hazard ratio (95% CI), age, other sociodemographic and substance use disorder.		
Crump 2013b [41]	Colon	**HR 95% CI**		
		**Model 1** Men 1.84 (0.87, 3.86) Women 2.53 (1.31, 4.86)	**Model 2** Men 1.84 (0.87, 3.88) Women 2.34 (1.21, 4.51)	**Model 3** Men 1.87 (0.89, 3.95) Women 2.35 (1.22, 4.52)
		Model 1: HR, adjusted for age; Model 2: HR, adjusted for age and other sociodemographic variable (sociodemographic variables included marital status, education, employment status, and income); Model 3: HR, adjusted for age, other sociodemographic variables, and substance use disorders (substance use disorders included any outpatient or inpatient diagnosis of a substance use disorder)		
Perini 2014 [42]	Colon, rectum	**SMR 95% CI**		
		Schizophrenia 0.53 (0.05, 2.51) Affective disorders 0.88 (0.46, 1.57) Neurosis 1.48 (0.66, 2.92) Personality disorders 0		
Cunningham 2015 [43]	Colorectal	**HR 95% CI**		
		**Model 1** SMI 2.84 (1.70, 4.73) AMI 1.21 (0.88, 1.67)	**Model 3** SMI 2.17 (1.30, 3.63) AMI 1.47 (1.07, 2.04)	**Model 5** SMI 1.89 (1.12, 3.17) AMI 1.25 (0.89, 1.75)
		**Model 2** SMI 2.94 (1.75, 4.87) AMI 1.15 (0.84, 1.59)	**Model 4** SMI 2.01 (1.20, 3.36) AMI 1.47 (1.06, 2.03)	
		Model 1 =crude survival; Model 2 =adj for age+sex+ethnicity; Model 3=1+SEER stage at diagnosis; Model 4 =2+NZ Deprivation Index score; Model 5=3+C3 Comorbidity Index score.		
Olfson 2015 [44]	Colon	**MR per 100 000 person-years.**		
		**Gender** Total 14.1 Men 13.1 Women 15.3	**Age groups** 20-34 yrs. 0.8 35-54 yrs. 8.6 55-64 yrs. 46.6	**Ethnicity** White Non-Hispanic 16.4 Black Non-Hispanic 13.3 Other Non-Hispanic 5.9 Hispanic 8
		**SMR 95% CI**		
		**Gender** Total 1.7 (1.6, 1.8) Men 1.6 (1.4, 1.8) Women 1.9 (1.7, 2.1)	**Age groups** 20-34 yrs. 1.5 (0.5, 2.6) 35-54 yrs. 1.4 (1.2, 1.6) 55-64 yrs. 2 (1.8, 2.2)	**Ethnicity** White Non-Hispanic 2.2 (2, 2.4) Black Non-Hispanic 1.1 (1, 1.3) Other Non-Hispanic 1.3 (0.5, 2.1) Hispanic 1.6 (0.9, 2.4)
Sun 2015 [45]	Colorectal	**Crude MRR** AD 1-4m 1.94 AD 5-8m 1.50 AD 9-12m 1.97 AD 13-16m 1.74 AD 17-20m 1.60 AD 21-24m 2.18 AD ≥25m 1.65	**aMRR (95% CI)** AD 1-4m 1.57 (1.34, 1.83) AD 5-8m 1.12 (0.86, 1.47) AD 9-12m 1.51 (1.16, 1.96) AD 13-16m 1.29 (0.97, 1.72) AD 17-20m 1.15 (0.85, 1.56) AD 21-24m 1.74 (1.31, 2.31) AD ≥25m 1.29 (1.18, 1.41)	
		Figure 2: MRR the first year after cancer diagnosis according to the time of antidepressant (AD) treatment initiation before cancer diagnosis for current AD users diagnosed with different types of cancer, including CRC. Adjusted for sex, age at time of diagnosis, stage of cancer, Charlson comorbidity index, marital status, education, and calendar year		
Manderbacka 2018 [46]	Colorectal	**HR 95% CI**		
		**Model 1** Men Psychosis 1.58 (1.33–1.86) Mood disorder 1.06 (0.87–1.30) Women Psychosis 1.54 (1.34–1.76) Mood disorder 1.11 (0.96–1.28)	**Model 2** Men Psychosis 1.73 (1.46–2.04) Mood disorder 1.06 (0.87–1.30) Women Psychosis 1.44 (1.26–1.64) Mood disorder 1.12 (0.97–1.29)	**Model 3** Men Psychosis 1.72 (1.46–2.04) Mood disorder 1.11 (0.91–1.36) Women Psychosis 1.37 (1.20–1.57) Mood disorder 1.10 (0.95–1.27)
		**Five-year cancer mortality**		
		**1990-1994 HR 95% CI** Men Psychosis 1.77 (1.11–2.82) Mood disorder 0.91 (0.49–1.70) Women Psychosis 1.16 (0.79–1.69) Mood disorder 1.34 (0.86–2.08)	**1997-2001 HR 95% CI** Men Psychosis 1.79 (1.26–2.55) Mood disorder 0.98 (0.59–1.63) Women Psychosis 1.39 (1.06–1.83) Mood disorder 0.85 (0.61–1.16)	**2004-2008 HR 95% CI** Men Psychosis 1.74 (1.17–2.58) Mood disorder 1.62 (1.13–2.32) Women Psychosis 1.77 (1.34–2.34) Mood disorder 1.01 (0.74–1.38)
		People without a history of SMI are used as the reference group. Model 1: controlling for age, year, cancer topography and morphology and comorbidities. Model 2: Model 1 stage at presentation. Model 3: Model 2 cancer treatment.		
Toender 2018 [47]	Colorectal	**MR per 100 PYR 95% CI**		**MRR 95% CI**
		SMI Total 22.70 (19.50–24.40) Men 23.87 (20.96–27.19) Women 21.80 (19.48–24.39)	Not SMI Total 16.30 (16.20–16.50) Men 17.53 (17.35–17.69) Women 15.22 (15.07–15.38)	Total 1.39 (1.13–1.39) Men 1.36 (1.12–1.36) Women 1.43 (1.15–1.43)
		Unadjusted mortality rate after incident cancer diagnosis per 100 person-years (age as underlying time scale).		
Klaassen 2019 [48]	Colorectal	**HR 95% CI**		
		PUG 1 Outpatient 1.04 (1.02–1.07) PUG 2 ER visit 1.15 (1.01–1.31) PUG 3 Inpatient 1.71 (1.47–1.99)		
		All models adjusted for age at diagnosis, ADG comorbidity, income quintile, rurality, year of diagnosis. Model also adjusted for gender. PUG – psychiatric utilization gradient.		
Harris 2020 [49]	Colorectal	**5-year cumulative mortality *p*-value**		
		Figure S4 (Cancer-specific mortality) 5-year incidence of 20% in the control group vs. 21% in the SMI groups, *p* < 0.0001		
Liang 2020 [60]	Colorectal	**HR 95% CI**		
		**Model 1** Depression 0.90 (0.71, 1.14) Depression symp. 0.90 (0.68, 1.19) Depression AD 0.79 (0.52, 1.18)	**Model 2** Localized cancer Depression 0.82 (0.41. 1.64) Depression symp. 0.87 (0.41, 1.87) Depression AD 0.89 (0.68, 1.16)	Regional or distant cancer Depression 0.89 (0.68, 1.16) Depression symp. 0.93 (0.69, 1.26) Depression AD 0.79 (0.50, 1.23)
		Model 1: HR, age-adjusted; Model 2: HR, results were adjusted for age at baseline, race/ethnicity, body mass index (BMI), smoking, physical activity, total energy intake, dietary fiber, percent calories from fat, comorbidity at baseline, family history of colorectal cancer mortality, receipt of colonoscopy, NSAIDs use, history of postmenopausal hormone use, tumor stage, and treatment arm in each CT		
Mahar 2020, 2021 [50, 51]	Colorectal	**HR 95% CI**	**Absolute survival (excess deaths**	
		**Model 1** Outpatient 1.12 (0.93, 1.33) Inpatient 1.51 (1.22, 1.87) **Model 2** Outpatient 1.24 (1.04, 1.48) Inpatient 1.69 (1.36, 2.09)	**Model 1 parameter estimate 95% CI *p*-value** Outpatient 0.021 (0.0053, 0.037) 0.014 Inpatient 0.068 (0.039, 0.097) < 0.001	**Model 2 parameter estimate 95% CI *p*-value** Outpatient 0.033 (0.017, 0.049) < 0.001 Inpatient 0.082 (0.055, 0.11) < 0.001
		Model 1: crude; Model 2: Adjusted for age, sex, rurality, year of diagnosis, and tumor location		
Grassi 2021 [52]	Colon, rectosigmoid junction, rectum, anus	**SMR 95% CI**		
		**Overall** SMD 1.42 (0.92, 2.09) Schizophrenia+ 1.28 (0.70, 2.14) Bipolar+ 1.65 (0.82, 2.95)	**Men** SMD 1.26 (0.60, 2.31) Schizophrenia+ 1.26 (0.46, 2.74) Bipolar+ 1.25 (0.34, 3.20)	**Women** SMD 1.55 (0.87, 2.55) Schizophrenia+ 1.29 (0.56, 2.54) Bipolar+ 2.01 (0.81, 4.15)
Launders 2022 [53]	Bowel	**MR per 10,000 PYR 95% CI**	**MRR 95% CI**	**HR (95% CI) *p*-value**
		SMI 554.6 (430.59, 714.24) Not SMI 468.6 (411.56, 533.55)	1.18 (1.14, 2.28)	**Model 1** SMI 1.39 (0.85, 2.28) 0.188 **Model 2** SMI 1.42 (0.84–2.39) 0.182
		Mortality rate per 10,000PYR; Model 1: Partially adjusted model: Adjusted for calendar year and sociodemographic factors (sex [except for breast and prostate cancer], primary care practice, ethnicity). Model B’2: Fully adjusted model: Additionally adjusted for smoking status (ever smoked) and BMI (underweight [< 18.5], normal [18.5–24.9], overweight [25–29.9], obese [≥ 30]).		
Cheng 2023 [54]	Colon, rectum	**MR (years at risk not reported)**	**MRRs 95% CI**	**MIRs**
		Not SMI 21.4 Schizophrenia 27.4	1.28 (1.12, 1.47) *p* < 0.001	Controls 0.33 Cases 0.50
Chierzi 2023 [55]	Colon, rectosigmoid junction, rectum, anus	**SMR 95% CI**		
		**Overall** CMD 1.89 (1.68, 2.13) Depression 2.15 (1.87, 2.46) Neurotic disorders 1.44 (1.13, 1.79)	**Men** CMD 2.14 (1.80, 2.52) Depression 2.25 (1.82, 2.76) Neurotic disorders 1.96 (1.45, 2.58)	**Women** CMD 1.70 (1.44, 2.00) Depression 2.07 (1.71, 2.48) Neurotic disorders 0.99 (0.66, 1.42)
Oh 2023 [56]	Colorectal	**30-day postoperative mortality**		
		Adjusted OR (95% CI) *p*-value 1.12 (0.90, 1.40) 0.303		
		Multivariable logistic regression model: Age and sex, SES-related information, preoperative CCI score and disability, types of cancer and year of surgery; Subgroup analyses: type of cancer surgery, age, sex, and preoperative CCI		
Drevinskaite 2024 [58]	Colon, rectum, rectosigmoid, anus	**SMR 95% CI**		
		**Overall** Total 0.80 (0.50, 1.20) Men 0.74 (0.40, 1.26) Women 0.93 (0.53, 1.52)	**C18 (colon cancer)** Men 0.37 (0.12, 1.15) Women 0.88 (0.44, 1.77)	**C19-C21** (rectosigmoid junction, rectum and anus cancers) Men 1.11 (0.58, 2.13) Women 0.85 (0.38, 1.90)
Wootten 2024 [58]	Colorectal	**HR 95% CI**		
		**Model 1** 1.65 (1.35, 2.01)		
		Model 1: adjusted for age, sex, neighborhood-level income quintile, rurality of residence, access to a family physician, and comorbidities		

ACM: all-cause mortality; AD: antidepressants; AMI: any mental illness; C18: colon cancer; C19-C21: rectum, rectosigmoid, anus cancer cancers; C18-C21: colon, rectum, rectosigmoid, anus cancers; CMD: common mental disorders; CSM: cancer-specific mortality; CSS: cancer-specific survival; HR: hazard ratio; OR: odds ratio; OS: overall survival; MIR: mortality-to-incidence ratios; MR: mortality rate; MRR: mortality rate ratio; PD: psychiatric disorder; PUG: psychiatric utilization gradient; SMD: severe mental disorder; SMI: severe mental illness; SMR: standardized mortality ratios.

### Critical appraisal

The STROBE Statement was first published in 2007 [27]. A total of 22 of 24 studies included were published in or after 2007; however, only four of these studies utilized the STROBE guidelines [48, 50, 56, 58].

### Synthesis of results

#### Narrative synthesis of results

Overall, most studies reported increased CRC mortality for persons with SMI, when compared with either a control group or the general population without SMI.

Six studies [38, 47, 50, 53, 54, 60] reported CRC-cases and CRC-deaths in both the SMI group and the control group without SMI. In five of these studies [38, 47, 50, 53, 54], the estimated case fatality rates were higher for the SMI group compared with the corresponding control group. The lowest estimated case fatality was 16% in the SMI group and 12% in the corresponding control group [53]. The highest estimated case fatality was 81% in the SMI group and 77% in the corresponding control group [47] (Supplementary file 7).

Three studies [47, 53, 54] reported MRs for the SMI group and the control group. The corresponding MRRs ranged from 1.18 (1.14 to 1.23) [53] over a 19-year period to 1.39 (1.13 to 1.70) [47] over a 36-year period, indicating an increased mortality of between 18 and 39% (Supplementary file 8).

Eight studies [34, 35, 42, 44, 52, 55, 57, 59] reported SMRs (95% CI) for mixed SMI groups, and the results ranged from 0.90 (0.72 to 1.12) [34] to 1.89 (1.68 to 2.13) [55]. Cumulative estimates of observed and expected deaths showed an excess of 414 CRC-deaths in the SMI group compared to the general population (Supplementary file 9).

Ten studies [39–41, 43, 46, 48, 50, 53, 58, 60] reported HRs (95% CI) for CRC-death, and estimates ranged from 0.90 (0.71 to 1.14) in a cohort of postmenopausal women with depression [60] to 2.35 (1.22 to 4.52) in a group of women with schizophrenia [41] (Supplementary file 10).

Finally, one study reported an odds ratio (OR, 95% CI) for 30-day mortality following surgery for CRC at 1.12 (0.90 to 1.40) [56].

### Meta-analyses of results

**Figure 2:**
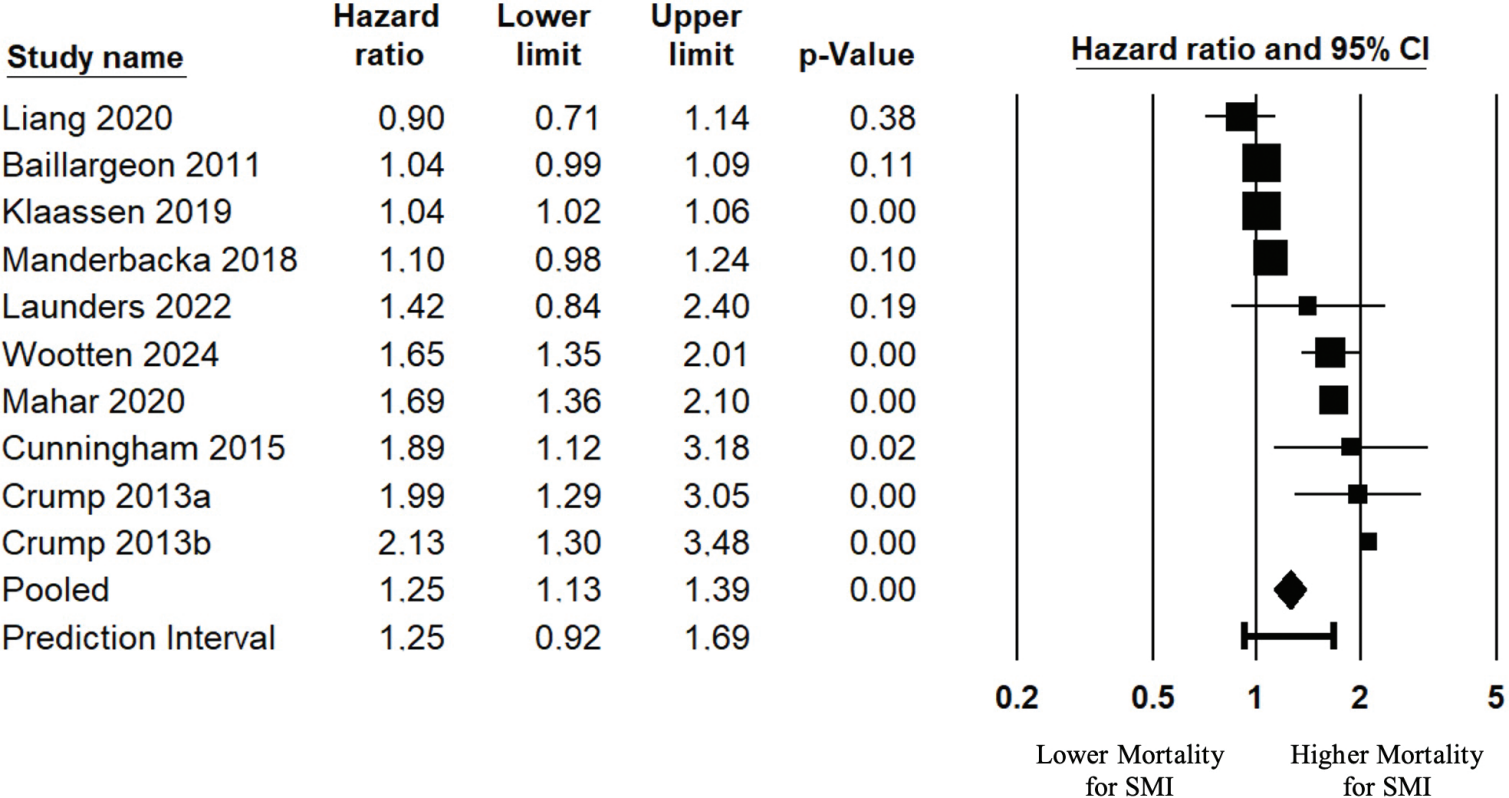
Colorectal cancer mortality hazard ratio, pooled analysis. Only fully adjusted HR-estimates were included in this analysis. Complete information about model adjustment is available in Table 2. Risk estimate.

When pooling the HRs of 10 studies [39–41, 43, 46, 48, 50, 53, 58, 60] with a total of 13,178,161 participants, persons with SMI had a 25% increased CRC mortality (95% CI: 1.13 to 1.39), compared with controls without SMI (Figure 2). When adjusting for possible publication bias, the estimate was reduced to an 18% increased mortality (95% CI: 1.06 to 1.30) (Table 3).

**Table 3:** Pooled analysis.

Outcome	Heterogeneity					Pooled effect size estimate			
	K^a^	N^b^	Q^c^	*P*	*I*^2^ ^d^	ES^e^	95% CI	*P*	95% PI^f^
Hazard Ratio (HR)^g^	10	13,178,161	64.7	< 0.001	86.1	1.25	1.13–1.39	< 0.001	0.92–1.69
Hazard Ratio (HR) (adjusted)^h^	(13)	-	-	-	-	1.18	1.06–1.30	*-*	-
Standardized Mortality Rate (SMR)^i^	8	1,373,819	61.6	< 0.001	88.6	1.38	1.08–1.77	0.010	0.63–3.03
Relative Risk (RR): Mortality^j^	8	2,723,332	55.6	< 0.001	87.4	1.11	1.04–1.19	0.004	0.88–1.40

CI: confidence interval.

a K = number of studies/independent samples in the analysis.

b Total number of participants in the analysis.

c Q statistic: *P* values <0.1 taken to suggest heterogeneity.

d *I*^2^ statistic: the proportion of the variance explained by differences in effect sizes beyond random error (heterogeneity).

e Pooled effect size (random effects model), that is, HR, SMR, or RR.

f 95% Prediction interval (PI), that is, the interval in which 95% of future observations from the same family of studies will fall, given the observed data, calculated for heterogeneous ESs (*I*^2^ > 0).

g Pooled results of longitudinal studies presenting the risk of mortality associated with severe mental illness (SMI) as hazard ratios (HRs). The total N is based on reported 12,724,7007 (3 studies) and 453,454 SMI with CRC (7 studies).

h For pooled estimates from K ≥ 10, the possibility of publication bias was explored with funnel plots and Egger’s test. ‘Missing studies’ were imputed with the Duval & Tweedie trim and fill method and the adjusted ES calculated.

i Pooled results of studies presenting the risk of cancer-related mortality associated with SMI as standardized mortality rates (SMRs), that is, mortality compared to expected mortality.

j Results of studies presenting data on the mortality of CRC patients with and without SMI. The ES is calculated as the relative risk (RR) of death associated with having SMI.

**Figure 3:**
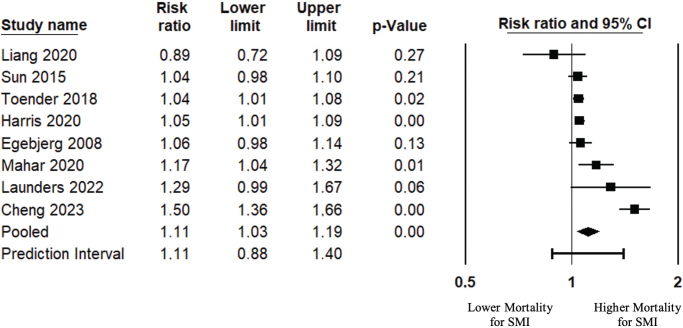
Colorectal cancer mortality risk ratio in SMI group compared with the control group, pooled analysis. Mortality risk ratio was assessed using the reported sample sizes and events (CRC deaths) for both the SMI group and the control group.

When pooling the results of the eight studies [38, 45, 47, 49, 50, 53, 54, 60] providing sample sizes and mortality event data to calculate an estimate of RRs, individuals with CRC and SMI were 11% (95% CI: 1.04 to 1.19) more likely to die from CRC than individuals with CRC but without SMI (Figure 3, Table 3).

**Figure 4:**
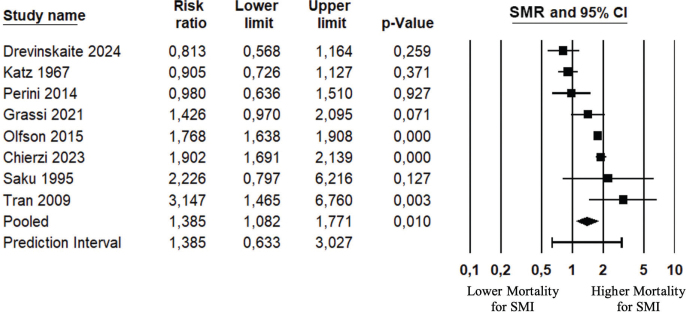
Standardized mortality ratio, pooled analysis. The standardized mortality ratio (SMR) is the ratio between the number of deaths observed in the SMI group and the number of expected deaths estimated from the reference population. SMR = 1 indicates that the number of observed deaths is equal to the number of expected deaths. SMR < 1 indicates fewer observed deaths than expected deaths. SMR > 1 indicates more observed deaths than expected deaths, and the difference between observed deaths and expected deaths is also termed excess deaths.

As seen in Figure 4 and Table 3, the pooled SMR-estimate was 38% (95% CI: 1.08 to 1.77) higher than expected based on the reference populations (eight studies [34, 35, 42, 44, 52, 55, 57, 59], *n* = 2,723,332 with SMI).

The pooled HRs, RRs, and SMR-ratios were characterized by a high degree of heterogeneity, indicating that large proportions of the between-study differences in ESs are caused by systematic differences between study characteristics, rather than sampling error (Table 3). While this suggests the need for analysis of moderators, for example, mental illness severity, sex, etc., the number of studies per moderator was too small for robust analyses [61].

## Discussion

In this scoping review, we identified 24 original studies, published between 1967 and 2024, investigating CRC mortality in persons with SMI. Our findings suggest that persons with SMI experienced increased mortality following CRC diagnosis, no matter how these studies were conducted. Similar cancer outcomes have been reported for persons with schizophrenia, that is, increased mortality following breast cancer by 48–97% (RR: 1.48 [1.01 to 2.16] [62]; RR: 1.97 [1.38 to 2.83] [63]), lung cancer by 93% (RR: 1.93 [1.46 to 2.54] [63]), and pancreas cancer by 40% (RR: 1.40 [1.21 to 1.62]) [62]. By comparison, our pooled estimate for the entire SMI population, including schizophrenia, which is one of the most SMI, is more modest.

There are various hypotheses that could explain the observed inequity, that is, ‘systematic differences in health status or in the distribution of health resources’ [64], that result in worse CRC outcomes for persons with SMI. On the one hand, health inequity could be associated with personal factors, such as diet, smoking status, and physical activity level [2]. On the other hand, this inequity could be related to disorder-specific factors, such as positive, negative, and cognitive symptom burden; disorder prognosis; number of relapses; service utilization; adherence to treatment plan; side-effects of antipsychotics; and increased morbidity, which are compounded by stigma and economic deprivation associated with SMI [40, 41, 54, 65]. These micro-level determinants of health can further impact the person’s ability to notice the first symptoms of CRC, seek medical care, communicate symptoms accurately, and actively participate in the diagnostic, treatment, and follow-up process [65].

Similarly, provider and healthcare system factors can increase health inequity. These include stereotypes associated with SMI [65], access to primary care and CRC screening programs, prohibitive healthcare service costs, and receipt of nonstandard treatment. These factors linked to the subnational healthcare system characterized by local/regional density, accessibility, availability, culture, and practices are termed ‘meso-level’ [66] determinants and have been found to result in more advanced CRC-stage at diagnosis, lower likelihood of consultation with a specialist oncologist, lower probability of receiving cancer treatment, and higher health expenditures and out-of-pocket costs [19, 39, 49, 50, 58]. In other words, internalized stigma, and interactional and structural discrimination can interfere with a person seeking or receiving adequate treatment for CRC.

Finally, persons with SMI experience barriers to physical health care because at the macro-level in most healthcare systems, physical health and mental health are disconnected, assigned to insular healthcare systems, with no overlap of care [67].

A variety of top-down and bottom-up approaches are aimed at improving the physical health of persons with SMI. The Core20PLUS5 [68] initiative aims to reduce healthcare inequalities at the national level by ensuring that persons with SMI access annual physical health checks. CRC-specific initiatives include aggregating cancer screenings into a three-in-one appointment, where CRC and cervical screening are combined with breast cancer screening [69], and moving CRC screening closer to the most vulnerable persons with SMI, that is, on the Psychiatric Inpatient Unit [70].

Due to the high diagnostic heterogeneity of SMI cohorts, study design, choice of registers, and inclusion criteria, we were not able to assess the effects of age, sex, SMI subtype, severity, or service utilization on CRC mortality. Other studies have documented potential sex differences associated with SMI diagnosis and disease course [71]. For example, one Finish cohort showed that men with schizophrenia had higher mortality from suicide and cardiovascular disease, whereas women with schizophrenia had higher cancer mortality [71]. Similarly, one meta-analysis showed a more than two-fold higher risk of CRC mortality for women with schizophrenia (RR: 2.42 [1.39 to 4.22]), compared with 90% increased risk of CRC mortality for men with schizophrenia (RR: 1.90 [1.71 to 2.11]) [63]. While this suggests that women experience worse cancer-related outcomes, more work is needed to understand potential sex differences in CRC outcomes.

None of the studies included assessed functional impairment associated with SMI. For example, SAMHSA [12] distinguishes between depression alone and depression with severe impairment, where depression is assessed according to DSM-5, and the functional impairment is assessed using the Sheehan Disability Scale. Two studies in this review attempted to operationalize SMI severity by linking a clinical diagnosis to psychiatric service utilization [48, 50]. In these studies, persons requiring inpatient services had a 69–71% higher CRC mortality (HR: 1.69 [1.36 to 2.09]) to 1.71 [1.47 to 1.99]) [48, 50]. In contrast, persons receiving outpatient services had a 4–24% higher CRC mortality (HR: 1.04 [1.02 to 1.07] to 1.24 [1.04 to 1.48]) [48, 50]. However, in contrast, some of the earlier studies showed that long-term psychiatric hospitalization, longer than 10 years, was associated with lower cancer mortality, estimating 10% fewer deaths compared with the reference population with SMI [72]. It would appear that the institutional context could have both a protective and a detrimental effect on CRC outcomes.

### Strengths and limitations

This is the first review to comprehensively synthesize and quantify the inequity of CRC mortality for people with a wide spectrum of SMIs. We closely followed the *JBI Manual for Evidence Synthesis* [21] and *the PRISMA-ScR* guidelines [22] and registered an *a priori* protocol with Open Science Framework Registries to ensure the highest level of rigor and transparency. Second, we used well-defined criteria for study selection, we contacted and received additional data from corresponding authors, and we conducted state-of-the-art meta-analyses of these data using all effect estimates for CRC mortality (HRs, SMRs, and RRs based on population sample sizes).

A limitation to the scoping review methodology is that it does not include a quality assessment of the included studies. To account for this, we assessed adherence to *STROBE* [27] guidelines as documented within each included study. Second, the population of persons with SMI is well-defined neither in the literature nor clinically. Nevertheless, we defined SMI based on disorder-specific clinical diagnostic codes (ICD-10) by selecting disorders placed higher in this classification and cross-referencing these disorders with adult hospitalization reports and disability entitlement status reports. All included studies defined SMI based on clinical diagnosis using either the DSM or ICD-systems. By contrast, a diagnosis of SMI based on a screening questionnaire could constitute measurement bias due to faulty or imprecise diagnosis/symptom measurement [49, 73]. Furthermore, all studies provided adequate evidence that the SMI status/history preceded the CRC diagnosis, minimizing chronology bias [74].

There is an inherent limitation related to mortality data, which is typically obtained from death certificates. The cause of death reported on the death certificate may not accurately reflect the total contribution of a specific disease. Furthermore, mortality data could be skewed because of low rates of autopsies performed [75], regional differences, changing coding systems, and definitions of disease over time [76]. Our objective was to specifically describe and quantify differences in CRC outcomes for people with SMI, and we therefore defined CRC-specific mortality as our primary outcome. As such, we excluded all-cause mortality. It could be argued that all-cause mortality was an equally valid outcomee, as it would also include deaths resulting from CRC-complications [77]. However, cancer-specific mortality is the preferred end-point when investigating the effect of cancer screening and care programs, as this outcome can detect even small effects. Furthermore, CRC-specific mortality involves identifying only deaths that are due to CRC [78, 79].

Finally, we did not formulate context-specific exclusion criteria, and therefore, we did not use a time filter for our systematic search. The patient records spanned from 1955 to 2020 – a period that encompassed major transformations in medical sciences and healthcare services. A few examples include the restructuring of mental healthcare [80], development of the colonoscopy technique [81], discovery of second-generation anti-psychotics [82], publication of first CRC screening recommendations [83], and more recently roll-out of population-based CRC-screening programs [84]. We were not able to investigate differences over time in CRC mortality for persons with SMI. As such, knowledge about the impact of nationwide CRC-screening programs on equity of CRC outcomes for people with SMI is warranted.

## Conclusions

The evidence points consistently to an increased CRC mortality for persons with SMI compared with persons without SMI. Furthermore, this evidence supports the idea that persons with SMI are a heterogenous group, and as such, any future approach to improve CRC outcomes for persons with SMI would warrant a tailored approach to potentiate individual resources, to mitigate stigma and structural discrimination.

## Supplementary Material

Colorectal cancer mortality in persons with severe mental illness: a scoping review with meta-analyses of observational studies

## Data Availability

Data are available upon request to the corresponding author.
