# Supplementary material for: Colorectal cancer mortality in persons with severe mental illness: a scoping review with meta-analyses of observational studies
Source: Acta Oncol. 2025 Mar 5;64:42260. doi: 10.2340/1651-226X.2025.42260 (PMC11905152; doi:10.2340/1651-226X.2025.42260)
Supplement: Colorectal cancer mortality in persons with severe mental illness: a scoping review with meta-analyses of observational studies [file AO-64-42260-s1.pdf]

Supplementary material has been published as submitted. It has not been copyedited, or typeset by Acta Oncologica

Supplementary file 1: (PRISMA-ScR) Checklist

Reporting guidelines statement

Preferred Reporting Items for Systematic reviews and Meta-Analyses extension for Scoping Reviews (PRISMA-ScR) Checklist

SECTION	ITEM	PRISMA-ScR CHECKLIST ITEM	REPORTED ON PAGE #
**TITLE**			
Title	1	Identify the report as a scoping review.	1
**ABSTRACT**			
Structured summary	2	Provide a structured summary that includes (as applicable): background, objectives, eligibility criteria, sources of evidence, charting methods, results, and conclusions that relate to the review questions and objectives.	2
**INTRODUCTION**			
Rationale	3	Describe the rationale for the review in the context of what is already known. Explain why the review questions/objectives lend themselves to a scoping review approach.	3
Objectives	4	Provide an explicit statement of the questions and objectives being addressed with reference to their key elements (e.g., population or participants, concepts, and context) or other relevant key elements used to conceptualize the review questions and/or objectives.	4
**METHODS**			
Protocol and registration	5	Indicate whether a review protocol exists; state if and where it can be accessed (e.g., a Web address); and if available, provide registration information, including the registration number.	5
Eligibility criteria	6	Specify characteristics of the sources of evidence used as eligibility criteria (e.g., years considered, language, and publication status), and provide a rationale.	5-6, Suppl. File 2
Information sources*	7	Describe all information sources in the search (e.g., databases with dates of coverage and contact with authors to identify additional sources), as well as the date the most recent search was executed.	6
Search	8	Present the full electronic search strategy for at least 1 database, including any limits used, such that it could be repeated.	6, Suppl. Files 3, 4

SECTION	ITEM	PRISMA-ScR CHECKLIST ITEM	REPORTED ON PAGE #
Selection of sources of evidence†	9	State the process for selecting sources of evidence (i.e., screening and eligibility) included in the scoping review.	6
Data charting process‡	10	Describe the methods of charting data from the included sources of evidence (e.g., calibrated forms or forms that have been tested by the team before their use, and whether data charting was done independently or in duplicate) and any processes for obtaining and confirming data from investigators.	7
Data items	11	List and define all variables for which data were sought and any assumptions and simplifications made.	Suppl. File 5
Critical appraisal of individual sources of evidence§	12	If done, provide a rationale for conducting a critical appraisal of included sources of evidence; describe the methods used and how this information was used in any data synthesis (if appropriate).	7
Synthesis of results	13	Describe the methods of handling and summarizing the data that were charted.	7-8
**RESULTS**			
Selection of sources of evidence	14	Give numbers of sources of evidence screened, assessed for eligibility, and included in the review, with reasons for exclusions at each stage, ideally using a flow diagram.	9, Figure 1, Suppl. File 6
Characteristics of sources of evidence	15	For each source of evidence, present characteristics for which data were charted and provide the citations.	9-10
Critical appraisal within sources of evidence	16	If done, present data on critical appraisal of included sources of evidence (see item 12).	10
Results of individual sources of evidence	17	For each included source of evidence, present the relevant data that were charted that relate to the review questions and objectives.	Tables 1, 2
Synthesis of results	18	Summarize and/or present the charting results as they relate to the review questions and objectives.	11-12, Table 3, Figures 2-4, Suppl. Files 7-11
**DISCUSSION**			
Summary of evidence	19	Summarize the main results (including an overview of concepts, themes, and types of evidence available), link to the review questions and objectives, and consider the relevance to key groups.	13-15
Limitations	20	Discuss the limitations of the scoping review process.	15-16

SECTION	ITEM	PRISMA-ScR CHECKLIST ITEM	REPORTED ON PAGE #
Conclusions	21	Provide a general interpretation of the results with respect to the review questions and objectives, as well as potential implications and/or next steps.	17
**FUNDING**			
Funding	22	Describe sources of funding for the included sources of evidence, as well as sources of funding for the scoping review. Describe the role of the funders of the scoping review.	18

JBI = Joanna Briggs Institute; PRISMA-ScR = Preferred Reporting Items for Systematic reviews and Meta-Analyses extension for Scoping Reviews.

* Where *sources of evidence* (see second footnote) are compiled from, such as bibliographic databases, social media platforms, and Web sites.

† A more inclusive/heterogeneous term used to account for the different types of evidence or data sources (e.g., quantitative and/or qualitative research, expert opinion, and policy documents) that may be eligible in a scoping review as opposed to only studies. This is not to be confused with *information sources* (see first footnote).

‡ The frameworks by Arksey and O'Malley (6) and Levac and colleagues (7) and the JBI guidance (4, 5) refer to the process of data extraction in a scoping review as data charting.

§ The process of systematically examining research evidence to assess its validity, results, and relevance before using it to inform a decision. This term is used for items 12 and 19 instead of "risk of bias" (which is more applicable to systematic reviews of interventions) to include and acknowledge the various sources of evidence that may be used in a scoping review (e.g., quantitative and/or qualitative research, expert opinion, and policy document).

From: Tricco AC, Lillie E, Zarin W, O'Brien KK, Colquhoun H, Levac D, et al. PRISMA Extension for Scoping Reviews (PRISMAScR): Checklist and Explanation. Ann Intern Med. 2018;169:467–473. doi: 10.7326/M18-0850.

Supplementary file 2: Eligibility Criteria

	INCLUSION	EXCLUSION
**POPULATION**		
	Humans of adult age (≥18 years) with a clinical diagnosis of severe mental illness (SMI), or currently receiving treatment for SMI. SMI diagnosis precedes colorectal cancer (CRC) diagnosis. SMI is specified as (ICD-10):<br>• Schizophrenia, including psychosis (F20-29),<br>• Depression (F30-39),<br>• Anxiety, including PTSD (F40-49),<br>• Eating disorders (F50),<br>• Specific personality disorders (F60).<br>Any other mental disorders that are not mentioned above, can be included in the case of a heterogenous sample, if cases represent less than 5% of the total sample size.	Children or humans below the age of 18 years. SMI diagnosis following CRC diagnosis and/or treatment. Depression and/or anxiety onset, diagnosis and treatment as a result of CRC. Functional impairment due to other mental disorders, specified as (ICD-10):<br>• Dementia (F00-09),<br>• Substance abuse disorder (F10-19),<br>• Behavioral disturbances, except eating disorders (F51-59),<br>• Personality disorders, except specific personality disorders (F61-69),<br>• Cognitive impairments (F70-79),<br>• Developmental disorder (F80-89),<br>• Behavioral and emotional disorders specific to children (F90-98),<br>• Unspecified mental disorders (F99)<br>SMI assessed by screening questionnaires, e.g., The Hospital Anxiety and Depression Scale (HADS), Symptom Checklist 90 (SCL-90-R).
**CANCER TYPE**		
	Primary diagnosis of colorectal cancer (neoplasm), colon cancer, or rectal cancer.	Cancer metastases to the colon, rectum, or colorectal region. Cancer types of unknown primary site.
**COMPARATOR/CONTEXT**		
	Persons without mental illnesses diagnosed prior to CRC diagnosis<br>Persons without any SMI diagnosed prior to CRC diagnosis	Persons with any SMI diagnosis prior to CRC diagnosis<br>Persons with any SMI diagnosed simultaneously or following CRC diagnosis
**OUTCOME**		
	Primary outcomes: CRC mortality and survival, e.g., mortality rate, survival %, and additional effect estimates (HR, RR, SMR, MRR, etc).	All-cause mortality or mortality not due to CRC.
**STUDY TYPE**		
	Observational studies on SMI and mortality due to CRC: cross-sectional, cohort studies, longitudinal studies,	Review studies, RCTs, case studies or case reports, qualitative studies, conference abstracts, conference posters, theses/dissertations, and protocols.

prospective studies, retrospective studies, case-control studies.	Cross-sectional studies where prior SMI status to CRC diagnosis cannot be assessed

Supplementary file 3: Search Strategy in MEDLINE/PubMED

**Population**	**AND**	**Primary cancer type**	**AND**	**Outcome**
[MeSH terms]<br>"schizophrenia spectrum and other psychotic disorders", "schizophrenia", "psychotic disorders"<br>"mood disorders", "bipolar disorder", "depressive disorder, major", "depressive disorder"<br>"anxiety disorders", "anxiety"<br>"trauma and stressor related disorders", "stress disorders, post traumatic"<br>"feeding and eating disorders"<br>"personality disorders"		[MeSH Terms]<br>"colorectal neoplasms"<br>"colonic neoplasms"<br>"rectal neoplasms"		[MeSH Terms]<br>"mortality"<br>"survival"<br>"death"
**OR**		**OR**		**OR**
[text word]<br>schizophrenia spectrum and other psychotic disorders, Schizophrenia, Psychosis<br>Mood disorders, Bipolar disorder, Major depressive disorder<br>Anxiety disorder, Anxiety, PTSD<br>Eating disorder<br>Personality disorder<br>Severe mental illness, Serious mental illness, SMI		[text word]<br>Colorectal cancer<br>Colon cancer<br>Rectal cancer		[text word]<br>Mortality<br>Survival<br>Death

Search: ((((((((((((schizophrenia) OR (psychosis)) OR (bipolar disorder)) OR (major depressive disorder)) OR (anxiety)) OR (ptsd)) OR (eating disorders)) OR (personality disorders)) OR ((((((anxiety disorders) OR (feeding and eating disorders)) OR (mood disorders)) OR (personality disorders)) OR (schizophrenia spectrum and other psychotic disorders)) OR (trauma and stressor related disorders))) OR ((("severe mental illness") OR ("serious mental illness")) OR (SMI))) AND (((colorectal cancer) OR (colon cancer)) OR (rectal cancer))) AND (((mortality) OR (survival)) OR (death)) - saved search **Sort by:** most recent

Supplementary file 4: Search history in all databases

MEDLINE/PubMED

Search number	Query	Filters	Search Details	Results	Time
**1**	mortality		"mortality"[MeSH Terms] OR "mortality"[All Fields] OR "mortalities"[All Fields] OR "mortality"[MeSH Subheading]	1,587,216	06:34:22
**2**	survival		"mortality"[MeSH Subheading] OR "mortality"[All Fields] OR "survival"[All Fields] OR "survival"[MeSH Terms] OR "survivability"[All Fields] OR "survivable"[All Fields] OR "survivals"[All Fields] OR "survive"[All Fields] OR "survived"[All Fields] OR "survives"[All Fields] OR "surviving"[All Fields]	2,686,430	06:34:31
**3**	death		"death"[MeSH Terms] OR "death"[All Fields] OR "deaths"[All Fields]	1,248,478	06:34:42
**4**	colorectal cancer		"colorectal neoplasms"[MeSH Terms] OR ("colorectal"[All Fields] AND "neoplasms"[All Fields]) OR "colorectal neoplasms"[All Fields] OR ("colorectal"[All Fields] AND "cancer"[All Fields]) OR "colorectal cancer"[All Fields]	308,360	06:34:52
**5**	colon cancer		"colonic neoplasms"[MeSH Terms] OR ("colonic"[All Fields] AND "neoplasms"[All Fields]) OR "colonic neoplasms"[All Fields] OR ("colon"[All Fields] AND "cancer"[All Fields]) OR "colon cancer"[All Fields]	166,852	06:34:58
**6**	rectal cancer		"rectal neoplasms"[MeSH Terms] OR ("rectal"[All Fields] AND "neoplasms"[All Fields]) OR "rectal neoplasms"[All Fields] OR ("rectal"[All Fields] AND "cancer"[All Fields]) OR "rectal cancer"[All Fields]	84,842	06:35:07
**7**	mental disorders		"mental disorders"[MeSH Terms] OR ("mental"[All Fields] AND "disorders"[All Fields]) OR "mental disorders"[All Fields]	1,549,734	06:35:25

**8**	anxiety disorders		"anxiety disorders"[MeSH Terms] OR ("anxiety"[All Fields] AND "disorders"[All Fields]) OR "anxiety disorders"[All Fields]	180,746	06:35:56
**9**	feeding and eating disorders		"feeding and eating disorders"[MeSH Terms] OR ("feeding"[All Fields] AND "eating"[All Fields] AND "disorders"[All Fields]) OR "feeding and eating disorders"[All Fields]	40,283	06:36:16
**10**	mood disorders		"mood disorders"[MeSH Terms] OR ("mood"[All Fields] AND "disorders"[All Fields]) OR "mood disorders"[All Fields]	205,158	06:36:31
**11**	personality disorders		"personality disorders"[MeSH Terms] OR ("personality"[All Fields] AND "disorders"[All Fields]) OR "personality disorders"[All Fields]	88,187	06:36:46
**12**	schizophrenia spectrum and other psychotic disorders		"schizophrenia spectrum and other psychotic disorders"[MeSH Terms] OR ("schizophrenia"[All Fields] AND "spectrum"[All Fields] AND "other"[All Fields] AND "psychotic"[All Fields] AND "disorders"[All Fields]) OR "schizophrenia spectrum and other psychotic disorders"[All Fields]	166,017	06:37:09
**14**	trauma and stressor related disorders		"trauma and stressor related disorders"[MeSH Terms] OR ("trauma"[All Fields] AND "stressor"[All Fields] AND "related"[All Fields] AND "disorders"[All Fields]) OR "trauma and stressor related disorders"[All Fields]	51,930	06:37:52
**15**	schizophrenia		"schizophrenia"[MeSH Terms] OR "schizophrenia"[All Fields] OR "schizophrenias"[All Fields] OR "schizophrenia s"[All Fields]	166,899	06:38:05
**16**	bipolar disorder		"bipolar disorder"[MeSH Terms] OR ("bipolar"[All Fields] AND "disorder"[All Fields]) OR "bipolar disorder"[All Fields]	62,289	06:38:20
**17**	major depressive disorder		"depressive disorder, major"[MeSH Terms] OR ("depressive"[All Fields] AND "disorder"[All Fields] AND "major"[All Fields]) OR "major depressive disorder"[All Fields] OR ("major"[All	158,221	06:38:32

			Fields] AND "depressive"[All Fields] AND "disorder"[All Fields]) OR "depressive disorder"[MeSH Terms] OR ("depressive"[All Fields] AND "disorder"[All Fields]) OR "depressive disorder"[All Fields]		
**18**	psychosis		"psychotic disorders"[MeSH Terms] OR ("psychotic"[All Fields] AND "disorders"[All Fields]) OR "psychotic disorders"[All Fields] OR "psychosis"[All Fields]	100,274	06:38:42
**21**	eating disorders		"feeding and eating disorders"[MeSH Terms] OR ("feeding"[All Fields] AND "eating"[All Fields] AND "disorders"[All Fields]) OR "feeding and eating disorders"[All Fields] OR ("eating"[All Fields] AND "disorders"[All Fields]) OR "eating disorders"[All Fields]	55,219	06:39:48
**22**	ptsd		"stress disorders, post traumatic"[MeSH Terms] OR ("stress"[All Fields] AND "disorders"[All Fields] AND "post traumatic"[All Fields]) OR "post-traumatic stress disorders"[All Fields] OR "ptsd"[All Fields]	57,714	06:40:08
**25**	"severe mental illness"		"severe mental illness"[All Fields]	5,890	06:40:43
**26**	"serious mental illness"		"serious mental illness"[All Fields]	4,853	06:40:50
**27**	Mental illness		"mental disorders"[MeSH Terms] OR ("mental"[All Fields] AND "disorders"[All Fields]) OR "mental disorders"[All Fields] OR ("mental"[All Fields] AND "illness"[All Fields]) OR "mental illness"[All Fields]	1,575,634	06:41:22
**28**	((mortality) OR (survival)) OR (death)		"mortality"[MeSH Terms] OR "mortality"[All Fields] OR "mortalities"[All Fields] OR "mortality"[MeSH Subheading] OR "mortality"[MeSH Subheading] OR "mortality"[All Fields] OR "survival"[All Fields] OR "survival"[MeSH Terms] OR "survivability"[All Fields] OR "survivable"[All Fields] OR "survivals"[All Fields] OR "survive"[All Fields] OR "survived"[All Fields]	3,493,542	06:42:05

			OR "survives"[All Fields] OR "surviving"[All Fields] OR "death"[MeSH Terms] OR "death"[All Fields] OR "deaths"[All Fields]		
**29**	((colorectal cancer) OR (colon cancer)) OR (rectal cancer)		"colorectal neoplasms"[MeSH Terms] OR ("colorectal"[All Fields] AND "neoplasms"[All Fields]) OR "colorectal neoplasms"[All Fields] OR ("colorectal"[All Fields] AND "cancer"[All Fields]) OR "colorectal cancer"[All Fields] OR ("colonic neoplasms"[MeSH Terms] OR ("colonic"[All Fields] AND "neoplasms"[All Fields]) OR "colonic neoplasms"[All Fields] OR ("colon"[All Fields] AND "cancer"[All Fields]) OR "colon cancer"[All Fields]) OR ("rectal neoplasms"[MeSH Terms] OR ("rectal"[All Fields] AND "neoplasms"[All Fields]) OR "rectal neoplasms"[All Fields] OR ("rectal"[All Fields] AND "cancer"[All Fields]) OR "rectal cancer"[All Fields])	364,240	06:42:22
**30**	(((((anxiety disorders) OR (feeding and eating disorders)) OR (mood disorders)) OR (personality disorders)) OR (schizophrenia spectrum and other psychotic disorders)) OR (trauma and stressor related disorders)		"anxiety disorders"[MeSH Terms] OR ("anxiety"[All Fields] AND "disorders"[All Fields]) OR "anxiety disorders"[All Fields] OR ("feeding and eating disorders"[MeSH Terms] OR ("feeding"[All Fields] AND "eating"[All Fields] AND "disorders"[All Fields]) OR "feeding and eating disorders"[All Fields]) OR ("mood disorders"[MeSH Terms] OR ("mood"[All Fields] AND "disorders"[All Fields]) OR "mood disorders"[All Fields]) OR ("personality disorders"[MeSH Terms] OR ("personality"[All Fields] AND "disorders"[All Fields]) OR "personality disorders"[All Fields]) OR ("schizophrenia spectrum and other psychotic disorders"[MeSH Terms] OR ("schizophrenia"[All Fields] AND "spectrum"[All Fields] AND "other"[All Fields] AND "psychotic"[All Fields] AND "disorders"[All Fields]) OR "schizophrenia spectrum and other psychotic disorders"[All Fields]) OR ("trauma and stressor related	600,680	06:44:22

			disorders"[MeSH Terms] OR ("trauma"[All Fields] AND "stressor"[All Fields] AND "related"[All Fields] AND "disorders"[All Fields]) OR "trauma and stressor related disorders"[All Fields])		
**32**	(("severe mental illness") OR ("serious mental illness")) OR (SMI)		"severe mental illness"[All Fields] OR "serious mental illness"[All Fields] OR "SMI"[All Fields]	18,171	06:47:47
**33**	(((("severe mental illness") OR ("serious mental illness")) OR (SMI)) AND (((colorectal cancer) OR (colon cancer)) OR (rectal cancer))) AND (((mortality) OR (survival)) OR (death))		("severe mental illness"[All Fields] OR "serious mental illness"[All Fields] OR "SMI"[All Fields]) AND ("colorectal neoplasms"[MeSH Terms] OR ("colorectal"[All Fields] AND "neoplasms"[All Fields]) OR "colorectal neoplasms"[All Fields] OR ("colorectal"[All Fields] AND "cancer"[All Fields]) OR "colorectal cancer"[All Fields] OR ("colonic neoplasms"[MeSH Terms] OR ("colonic"[All Fields] AND "neoplasms"[All Fields]) OR "colonic neoplasms"[All Fields] OR ("colon"[All Fields] AND "cancer"[All Fields]) OR "colon cancer"[All Fields]) OR ("rectal neoplasms"[MeSH Terms] OR ("rectal"[All Fields] AND "neoplasms"[All Fields]) OR "rectal neoplasms"[All Fields] OR ("rectal"[All Fields] AND "cancer"[All Fields]) OR "rectal cancer"[All Fields])) AND ("mortality"[MeSH Terms] OR "mortality"[All Fields] OR "mortalities"[All Fields] OR "mortality"[MeSH Subheading] OR ("mortality"[MeSH Subheading] OR "mortality"[All Fields] OR "survival"[All Fields] OR "survival"[MeSH Terms] OR "survivability"[All Fields] OR "survivable"[All Fields] OR "survivals"[All Fields] OR "survive"[All Fields] OR "survived"[All Fields] OR "survives"[All Fields] OR "surviving"[All Fields]) OR ("death"[MeSH Terms] OR "death"[All Fields] OR "deaths"[All Fields]))	90	06:48:11
**35**	(((((((anxiety disorders) OR (feeding and eating disorders))		("anxiety disorders"[MeSH Terms] OR ("anxiety"[All Fields] AND "disorders"[All	82	06:48:53

	OR (mood disorders)) OR (personality disorders)) OR (schizophrenia spectrum and other psychotic disorders)) OR (trauma and stressor related disorders)) AND (((colorectal cancer) OR (colon cancer)) OR (rectal cancer))) AND (((mortality) OR (survival)) OR (death))		Fields]) OR "anxiety disorders"[All Fields] OR ("feeding and eating disorders"[MeSH Terms] OR ("feeding"[All Fields] AND "eating"[All Fields] AND "disorders"[All Fields]) OR "feeding and eating disorders"[All Fields]) OR ("mood disorders"[MeSH Terms] OR ("mood"[All Fields] AND "disorders"[All Fields]) OR "mood disorders"[All Fields]) OR ("personality disorders"[MeSH Terms] OR ("personality"[All Fields] AND "disorders"[All Fields]) OR "personality disorders"[All Fields]) OR ("schizophrenia spectrum and other psychotic disorders"[MeSH Terms] OR ("schizophrenia"[All Fields] AND "spectrum"[All Fields] AND "other"[All Fields] AND "psychotic"[All Fields] AND "disorders"[All Fields]) OR "schizophrenia spectrum and other psychotic disorders"[All Fields]) OR ("trauma and stressor related disorders"[MeSH Terms] OR ("trauma"[All Fields] AND "stressor"[All Fields] AND "related"[All Fields] AND "disorders"[All Fields]) OR "trauma and stressor related disorders"[All Fields])) AND ("colorectal neoplasms"[MeSH Terms] OR ("colorectal"[All Fields] AND "neoplasms"[All Fields]) OR "colorectal neoplasms"[All Fields] OR ("colorectal"[All Fields] AND "cancer"[All Fields]) OR "colorectal cancer"[All Fields] OR ("colonic neoplasms"[MeSH Terms] OR ("colonic"[All Fields] AND "neoplasms"[All Fields]) OR "colonic neoplasms"[All Fields] OR ("colon"[All Fields] AND "cancer"[All Fields]) OR "colon cancer"[All Fields]) OR ("rectal neoplasms"[MeSH Terms] OR ("rectal"[All Fields] AND "neoplasms"[All Fields]) OR "rectal neoplasms"[All Fields] OR ("rectal"[All Fields] AND "cancer"[All Fields]) OR "rectal cancer"[All Fields])) AND ("mortality"[MeSH Terms] OR "mortality"[All Fields] OR "mortalities"[All		

			Fields] OR "mortality"[MeSH Subheading] OR ("mortality"[MeSH Subheading] OR "mortality"[All Fields] OR "survival"[All Fields] OR "survival"[MeSH Terms] OR "survivability"[All Fields] OR "survivable"[All Fields] OR "survivals"[All Fields] OR "survive"[All Fields] OR "survived"[All Fields] OR "survives"[All Fields] OR "surviving"[All Fields]) OR ("death"[MeSH Terms] OR "death"[All Fields] OR "deaths"[All Fields]))		
**42**	anxiety		"anxiety"[MeSH Terms] OR "anxiety"[All Fields] OR "anxieties"[All Fields] OR "anxiety s"[All Fields]	339,092	07:59:21
**43**	(((((((schizophrenia) OR (psychosis)) OR (bipolar disorder)) OR (major depressive disorder)) OR (anxiety)) OR (ptsd)) OR (eating disorders)) OR (personality disorders)		"schizophrenia"[MeSH Terms] OR "schizophrenia"[All Fields] OR "schizophrenias"[All Fields] OR "schizophrenia s"[All Fields] OR ("psychotic disorders"[MeSH Terms] OR ("psychotic"[All Fields] AND "disorders"[All Fields]) OR "psychotic disorders"[All Fields] OR "psychosis"[All Fields]) OR ("bipolar disorder"[MeSH Terms] OR ("bipolar"[All Fields] AND "disorder"[All Fields]) OR "bipolar disorder"[All Fields]) OR ("depressive disorder, major"[MeSH Terms] OR ("depressive"[All Fields] AND "disorder"[All Fields] AND "major"[All Fields]) OR "major depressive disorder"[All Fields] OR ("major"[All Fields] AND "depressive"[All Fields] AND "disorder"[All Fields]) OR "depressive disorder"[MeSH Terms] OR ("depressive"[All Fields] AND "disorder"[All Fields]) OR "depressive disorder"[All Fields]) OR ("anxiety"[MeSH Terms] OR "anxiety"[All Fields] OR "anxieties"[All Fields] OR "anxiety s"[All Fields]) OR ("stress disorders, post traumatic"[MeSH Terms] OR ("stress"[All Fields] AND "disorders"[All Fields] AND "post traumatic"[All Fields]) OR "post-traumatic stress	819,524	08:01:18

			disorders"[All Fields] OR "ptsd"[All Fields]) OR ("feeding and eating disorders"[MeSH Terms] OR ("feeding"[All Fields] AND "eating"[All Fields] AND "disorders"[All Fields]) OR "feeding and eating disorders"[All Fields] OR ("eating"[All Fields] AND "disorders"[All Fields]) OR "eating disorders"[All Fields]) OR ("personality disorders"[MeSH Terms] OR ("personality"[All Fields] AND "disorders"[All Fields]) OR "personality disorders"[All Fields])		
**44**	((((((((((schizophrenia) OR (psychosis)) OR (bipolar disorder)) OR (major depressive disorder)) OR (anxiety)) OR (ptsd)) OR (eating disorders)) OR (personality disorders)) OR ((((((anxiety disorders) OR (feeding and eating disorders)) OR (mood disorders)) OR (personality disorders)) OR (schizophrenia spectrum and other psychotic disorders)) OR (trauma and stressor related disorders))) OR ((("severe mental illness") OR ("serious mental illness")) OR (SMI))) AND (((colorectal cancer) OR (colon cancer)) OR (rectal cancer))) AND (((mortality) OR (survival)) OR (death))		("schizophrenia"[MeSH Terms] OR "schizophrenia"[All Fields] OR "schizophrenias"[All Fields] OR "schizophrenia s"[All Fields] OR ("psychotic disorders"[MeSH Terms] OR ("psychotic"[All Fields] AND "disorders"[All Fields]) OR "psychotic disorders"[All Fields] OR "psychosis"[All Fields]) OR ("bipolar disorder"[MeSH Terms] OR ("bipolar"[All Fields] AND "disorder"[All Fields]) OR "bipolar disorder"[All Fields]) OR ("depressive disorder, major"[MeSH Terms] OR ("depressive"[All Fields] AND "disorder"[All Fields] AND "major"[All Fields]) OR "major depressive disorder"[All Fields] OR ("major"[All Fields] AND "depressive"[All Fields] AND "disorder"[All Fields]) OR "depressive disorder"[MeSH Terms] OR ("depressive"[All Fields] AND "disorder"[All Fields]) OR "depressive disorder"[All Fields]) OR ("anxiety"[MeSH Terms] OR "anxiety"[All Fields] OR "anxieties"[All Fields] OR "anxiety s"[All Fields]) OR ("stress disorders, post traumatic"[MeSH Terms] OR ("stress"[All Fields] AND "disorders"[All Fields] AND "post traumatic"[All Fields]) OR "post-traumatic stress disorders"[All Fields] OR "ptsd"[All Fields]) OR ("feeding and eating disorders"[MeSH Terms] OR ("feeding"[All Fields] AND "eating"[All Fields] AND "disorders"[All Fields]) OR "feeding and	427	08:02:18

			eating disorders"[All Fields] OR ("eating"[All Fields] AND "disorders"[All Fields]) OR "eating disorders"[All Fields]) OR ("personality disorders"[MeSH Terms] OR ("personality"[All Fields] AND "disorders"[All Fields]) OR "personality disorders"[All Fields]) OR ("anxiety disorders"[MeSH Terms] OR ("anxiety"[All Fields] AND "disorders"[All Fields]) OR "anxiety disorders"[All Fields] OR ("feeding and eating disorders"[MeSH Terms] OR ("feeding"[All Fields] AND "eating"[All Fields] AND "disorders"[All Fields]) OR "feeding and eating disorders"[All Fields]) OR ("mood disorders"[MeSH Terms] OR ("mood"[All Fields] AND "disorders"[All Fields]) OR "mood disorders"[All Fields]) OR ("personality disorders"[MeSH Terms] OR ("personality"[All Fields] AND "disorders"[All Fields]) OR "personality disorders"[All Fields]) OR ("schizophrenia spectrum and other psychotic disorders"[MeSH Terms] OR ("schizophrenia"[All Fields] AND "spectrum"[All Fields] AND "other"[All Fields] AND "psychotic"[All Fields] AND "disorders"[All Fields]) OR "schizophrenia spectrum and other psychotic disorders"[All Fields]) OR ("trauma and stressor related disorders"[MeSH Terms] OR ("trauma"[All Fields] AND "stressor"[All Fields] AND "related"[All Fields] AND "disorders"[All Fields]) OR "trauma and stressor related disorders"[All Fields])) OR ("severe mental illness"[All Fields] OR "serious mental illness"[All Fields] OR "SMI"[All Fields])) AND ("colorectal neoplasms"[MeSH Terms] OR ("colorectal"[All Fields] AND "neoplasms"[All Fields]) OR "colorectal neoplasms"[All Fields] OR ("colorectal"[All Fields] AND "cancer"[All Fields]) OR "colorectal cancer"[All Fields] OR ("colonic neoplasms"[MeSH Terms] OR		

			("colonic"[All Fields] AND "neoplasms"[All Fields]) OR "colonic neoplasms"[All Fields] OR ("colon"[All Fields] AND "cancer"[All Fields]) OR "colon cancer"[All Fields]) OR ("rectal neoplasms"[MeSH Terms] OR ("rectal"[All Fields] AND "neoplasms"[All Fields]) OR "rectal neoplasms"[All Fields] OR ("rectal"[All Fields] AND "cancer"[All Fields]) OR "rectal cancer"[All Fields])) AND ("mortality"[MeSH Terms] OR "mortality"[All Fields] OR "mortalities"[All Fields] OR "mortality"[MeSH Subheading] OR ("mortality"[MeSH Subheading] OR "mortality"[All Fields] OR "survival"[All Fields] OR "survival"[MeSH Terms] OR "survivability"[All Fields] OR "survivable"[All Fields] OR "survivals"[All Fields] OR "survive"[All Fields] OR "survived"[All Fields] OR "survives"[All Fields] OR "surviving"[All Fields]) OR ("death"[MeSH Terms] OR "death"[All Fields] OR "deaths"[All Fields]))		
**45**	((((((((schizophrenia) OR (psychosis)) OR (bipolar disorder)) OR (major depressive disorder)) OR (anxiety)) OR (ptsd)) OR (eating disorders)) OR (personality disorders)) AND (((colorectal cancer) OR (colon cancer)) OR (rectal cancer))) AND (((mortality) OR (survival)) OR (death))		("schizophrenia"[MeSH Terms] OR "schizophrenia"[All Fields] OR "schizophrenias"[All Fields] OR "schizophrenia s"[All Fields] OR ("psychotic disorders"[MeSH Terms] OR ("psychotic"[All Fields] AND "disorders"[All Fields]) OR "psychotic disorders"[All Fields] OR "psychosis"[All Fields]) OR ("bipolar disorder"[MeSH Terms] OR ("bipolar"[All Fields] AND "disorder"[All Fields]) OR "bipolar disorder"[All Fields]) OR ("depressive disorder, major"[MeSH Terms] OR ("depressive"[All Fields] AND "disorder"[All Fields] AND "major"[All Fields]) OR "major depressive disorder"[All Fields] OR ("major"[All Fields] AND "depressive"[All Fields] AND "disorder"[All Fields]) OR "depressive disorder"[MeSH Terms] OR ("depressive"[All Fields] AND "disorder"[All Fields]) OR "depressive disorder"[All Fields]) OR	336	08:02:51

			("anxiety"[MeSH Terms] OR "anxiety"[All Fields] OR "anxieties"[All Fields] OR "anxiety s"[All Fields]) OR ("stress disorders, post traumatic"[MeSH Terms] OR ("stress"[All Fields] AND "disorders"[All Fields] AND "post traumatic"[All Fields]) OR "post-traumatic stress disorders"[All Fields] OR "ptsd"[All Fields]) OR ("feeding and eating disorders"[MeSH Terms] OR ("feeding"[All Fields] AND "eating"[All Fields] AND "disorders"[All Fields]) OR "feeding and eating disorders"[All Fields] OR ("eating"[All Fields] AND "disorders"[All Fields]) OR "eating disorders"[All Fields]) OR ("personality disorders"[MeSH Terms] OR ("personality"[All Fields] AND "disorders"[All Fields]) OR "personality disorders"[All Fields])) AND ("colorectal neoplasms"[MeSH Terms] OR ("colorectal"[All Fields] AND "neoplasms"[All Fields]) OR "colorectal neoplasms"[All Fields] OR ("colorectal"[All Fields] AND "cancer"[All Fields]) OR "colorectal cancer"[All Fields] OR ("colonic neoplasms"[MeSH Terms] OR ("colonic"[All Fields] AND "neoplasms"[All Fields]) OR "colonic neoplasms"[All Fields] OR ("colon"[All Fields] AND "cancer"[All Fields]) OR "colon cancer"[All Fields]) OR ("rectal neoplasms"[MeSH Terms] OR ("rectal"[All Fields] AND "neoplasms"[All Fields]) OR "rectal neoplasms"[All Fields] OR ("rectal"[All Fields] AND "cancer"[All Fields]) OR "rectal cancer"[All Fields])) AND ("mortality"[MeSH Terms] OR "mortality"[All Fields] OR "mortalities"[All Fields] OR "mortality"[MeSH Subheading] OR ("mortality"[MeSH Subheading] OR "mortality"[All Fields] OR "survival"[All Fields] OR "survival"[MeSH Terms] OR "survivability"[All Fields] OR "survivable"[All Fields] OR "survivals"[All Fields] OR "survive"[All Fields] OR "survived"[All Fields]		

			OR "survives"[All Fields] OR "surviving"[All Fields]) OR ("death"[MeSH Terms] OR "death"[All Fields] OR "deaths"[All Fields]))		
**46**	((((((((anxiety disorders) OR (feeding and eating disorders)) OR (mood disorders)) OR (personality disorders)) OR (schizophrenia spectrum and other psychotic disorders)) OR (trauma and stressor related disorders)) AND (((colorectal cancer) OR (colon cancer)) OR (rectal cancer))) AND (((mortality) OR (survival)) OR (death))) OR ((((("severe mental illness") OR ("serious mental illness")) OR (SMI)) AND (((colorectal cancer) OR (colon cancer)) OR (rectal cancer))) AND (((mortality) OR (survival)) OR (death)))) OR (((((((((schizophrenia) OR (psychosis)) OR (bipolar disorder)) OR (major depressive disorder)) OR (anxiety)) OR (ptsd)) OR (eating disorders)) OR (personality disorders)) AND (((colorectal cancer) OR (colon cancer)) OR (rectal cancer))) AND (((mortality) OR (survival)) OR (death)))		(("anxiety disorders"[MeSH Terms] OR ("anxiety"[All Fields] AND "disorders"[All Fields]) OR "anxiety disorders"[All Fields] OR ("feeding and eating disorders"[MeSH Terms] OR ("feeding"[All Fields] AND "eating"[All Fields] AND "disorders"[All Fields]) OR "feeding and eating disorders"[All Fields]) OR ("mood disorders"[MeSH Terms] OR ("mood"[All Fields] AND "disorders"[All Fields]) OR "mood disorders"[All Fields]) OR ("personality disorders"[MeSH Terms] OR ("personality"[All Fields] AND "disorders"[All Fields]) OR "personality disorders"[All Fields]) OR ("schizophrenia spectrum and other psychotic disorders"[MeSH Terms] OR ("schizophrenia"[All Fields] AND "spectrum"[All Fields] AND "other"[All Fields] AND "psychotic"[All Fields] AND "disorders"[All Fields]) OR "schizophrenia spectrum and other psychotic disorders"[All Fields]) OR ("trauma and stressor related disorders"[MeSH Terms] OR ("trauma"[All Fields] AND "stressor"[All Fields] AND "related"[All Fields] AND "disorders"[All Fields]) OR "trauma and stressor related disorders"[All Fields])) AND ("colorectal neoplasms"[MeSH Terms] OR ("colorectal"[All Fields] AND "neoplasms"[All Fields]) OR "colorectal neoplasms"[All Fields] OR ("colorectal"[All Fields] AND "cancer"[All Fields]) OR "colorectal cancer"[All Fields] OR ("colonic neoplasms"[MeSH Terms] OR ("colonic"[All Fields] AND "neoplasms"[All Fields]) OR "colonic neoplasms"[All Fields] OR ("colon"[All Fields] AND "cancer"[All Fields]) OR "colon cancer"[All Fields]) OR ("rectal neoplasms"[MeSH Terms] OR ("rectal"[All	427	08:03:30

			Fields] AND "neoplasms"[All Fields]) OR "rectal neoplasms"[All Fields] OR ("rectal"[All Fields] AND "cancer"[All Fields]) OR "rectal cancer"[All Fields])) AND ("mortality"[MeSH Terms] OR "mortality"[All Fields] OR "mortalities"[All Fields] OR "mortality"[MeSH Subheading] OR ("mortality"[MeSH Subheading] OR "mortality"[All Fields] OR "survival"[All Fields] OR "survival"[MeSH Terms] OR "survivability"[All Fields] OR "survivable"[All Fields] OR "survivals"[All Fields] OR "survive"[All Fields] OR "survived"[All Fields] OR "survives"[All Fields] OR "surviving"[All Fields]) OR ("death"[MeSH Terms] OR "death"[All Fields] OR "deaths"[All Fields]))) OR (("severe mental illness"[All Fields] OR "serious mental illness"[All Fields] OR "SMI"[All Fields]) AND ("colorectal neoplasms"[MeSH Terms] OR ("colorectal"[All Fields] AND "neoplasms"[All Fields]) OR "colorectal neoplasms"[All Fields] OR ("colorectal"[All Fields] AND "cancer"[All Fields]) OR "colorectal cancer"[All Fields] OR ("colonic neoplasms"[MeSH Terms] OR ("colonic"[All Fields] AND "neoplasms"[All Fields]) OR "colonic neoplasms"[All Fields] OR ("colon"[All Fields] AND "cancer"[All Fields]) OR "colon cancer"[All Fields]) OR ("rectal neoplasms"[MeSH Terms] OR ("rectal"[All Fields] AND "neoplasms"[All Fields]) OR "rectal neoplasms"[All Fields] OR ("rectal"[All Fields] AND "cancer"[All Fields]) OR "rectal cancer"[All Fields])) AND ("mortality"[MeSH Terms] OR "mortality"[All Fields] OR "mortalities"[All Fields] OR "mortality"[MeSH Subheading] OR ("mortality"[MeSH Subheading] OR "mortality"[All Fields] OR "survival"[All Fields] OR "survival"[MeSH Terms] OR "survivability"[All Fields] OR "survivable"[All Fields] OR "survivals"[All Fields] OR		

			"survive"[All Fields] OR "survived"[All Fields] OR "survives"[All Fields] OR "surviving"[All Fields]) OR ("death"[MeSH Terms] OR "death"[All Fields] OR "deaths"[All Fields]))) OR (("schizophrenia"[MeSH Terms] OR "schizophrenia"[All Fields] OR "schizophrenias"[All Fields] OR "schizophrenia s"[All Fields] OR ("psychotic disorders"[MeSH Terms] OR ("psychotic"[All Fields] AND "disorders"[All Fields]) OR "psychotic disorders"[All Fields] OR "psychosis"[All Fields]) OR ("bipolar disorder"[MeSH Terms] OR ("bipolar"[All Fields] AND "disorder"[All Fields]) OR "bipolar disorder"[All Fields]) OR ("depressive disorder, major"[MeSH Terms] OR ("depressive"[All Fields] AND "disorder"[All Fields] AND "major"[All Fields]) OR "major depressive disorder"[All Fields] OR ("major"[All Fields] AND "depressive"[All Fields] AND "disorder"[All Fields]) OR "depressive disorder"[MeSH Terms] OR ("depressive"[All Fields] AND "disorder"[All Fields]) OR "depressive disorder"[All Fields]) OR ("anxiety"[MeSH Terms] OR "anxiety"[All Fields] OR "anxieties"[All Fields] OR "anxiety s"[All Fields]) OR ("stress disorders, post traumatic"[MeSH Terms] OR ("stress"[All Fields] AND "disorders"[All Fields] AND "post traumatic"[All Fields]) OR "post-traumatic stress disorders"[All Fields] OR "ptsd"[All Fields]) OR ("feeding and eating disorders"[MeSH Terms] OR ("feeding"[All Fields] AND "eating"[All Fields] AND "disorders"[All Fields]) OR "feeding and eating disorders"[All Fields] OR ("eating"[All Fields] AND "disorders"[All Fields]) OR "eating disorders"[All Fields]) OR ("personality disorders"[MeSH Terms] OR ("personality"[All Fields] AND "disorders"[All Fields]) OR "personality disorders"[All Fields])) AND		

			("colorectal neoplasms"[MeSH Terms] OR ("colorectal"[All Fields] AND "neoplasms"[All Fields]) OR "colorectal neoplasms"[All Fields] OR ("colorectal"[All Fields] AND "cancer"[All Fields]) OR "colorectal cancer"[All Fields] OR ("colonic neoplasms"[MeSH Terms] OR ("colonic"[All Fields] AND "neoplasms"[All Fields]) OR "colonic neoplasms"[All Fields] OR ("colon"[All Fields] AND "cancer"[All Fields]) OR "colon cancer"[All Fields]) OR ("rectal neoplasms"[MeSH Terms] OR ("rectal"[All Fields] AND "neoplasms"[All Fields]) OR "rectal neoplasms"[All Fields] OR ("rectal"[All Fields] AND "cancer"[All Fields]) OR "rectal cancer"[All Fields])) AND ("mortality"[MeSH Terms] OR "mortality"[All Fields] OR "mortalities"[All Fields] OR "mortality"[MeSH Subheading] OR ("mortality"[MeSH Subheading] OR "mortality"[All Fields] OR "survival"[All Fields] OR "survival"[MeSH Terms] OR "survivability"[All Fields] OR "survivable"[All Fields] OR "survivals"[All Fields] OR "survive"[All Fields] OR "survived"[All Fields] OR "survives"[All Fields] OR "surviving"[All Fields]) OR ("death"[MeSH Terms] OR "death"[All Fields] OR "deaths"[All Fields])))		
**47**	personality disorder		"personality disorders"[MeSH Terms] OR ("personality"[All Fields] AND "disorders"[All Fields]) OR "personality disorders"[All Fields] OR ("personality"[All Fields] AND "disorder"[All Fields]) OR "personality disorder"[All Fields]	98,434	08:04:41
**48**	SMI		"SMI"[All Fields]	10,192	08:06:49
**49**	((((((((schizophrenia) OR (psychosis)) OR (bipolar disorder)) OR (major depressive disorder)) OR (anxiety)) OR (ptsd)) OR (eating disorders)) OR		"schizophrenia"[MeSH Terms] OR "schizophrenia"[All Fields] OR "schizophrenias"[All Fields] OR "schizophrenia s"[All Fields] OR ("psychotic disorders"[MeSH Terms] OR ("psychotic"[All Fields] AND "disorders"[All Fields]) OR "psychotic	881,072	08:18:24

	(personality disorders)) OR ((((((anxiety disorders) OR (feeding and eating disorders)) OR (mood disorders)) OR (personality disorders)) OR (schizophrenia spectrum and other psychotic disorders)) OR (trauma and stressor related disorders))) OR ((("severe mental illness") OR ("serious mental illness")) OR (SMI))		disorders"[All Fields] OR "psychosis"[All Fields]) OR ("bipolar disorder"[MeSH Terms] OR ("bipolar"[All Fields] AND "disorder"[All Fields]) OR "bipolar disorder"[All Fields]) OR ("depressive disorder, major"[MeSH Terms] OR ("depressive"[All Fields] AND "disorder"[All Fields] AND "major"[All Fields]) OR "major depressive disorder"[All Fields] OR ("major"[All Fields] AND "depressive"[All Fields] AND "disorder"[All Fields]) OR "depressive disorder"[MeSH Terms] OR ("depressive"[All Fields] AND "disorder"[All Fields]) OR "depressive disorder"[All Fields]) OR ("anxiety"[MeSH Terms] OR "anxiety"[All Fields] OR "anxieties"[All Fields] OR "anxiety s"[All Fields]) OR ("stress disorders, post traumatic"[MeSH Terms] OR ("stress"[All Fields] AND "disorders"[All Fields] AND "post traumatic"[All Fields]) OR "post-traumatic stress disorders"[All Fields] OR "ptsd"[All Fields]) OR ("feeding and eating disorders"[MeSH Terms] OR ("feeding"[All Fields] AND "eating"[All Fields] AND "disorders"[All Fields]) OR "feeding and eating disorders"[All Fields] OR ("eating"[All Fields] AND "disorders"[All Fields]) OR "eating disorders"[All Fields]) OR ("personality disorders"[MeSH Terms] OR ("personality"[All Fields] AND "disorders"[All Fields]) OR "personality disorders"[All Fields]) OR ("anxiety disorders"[MeSH Terms] OR ("anxiety"[All Fields] AND "disorders"[All Fields]) OR "anxiety disorders"[All Fields] OR ("feeding and eating disorders"[MeSH Terms] OR ("feeding"[All Fields] AND "eating"[All Fields] AND "disorders"[All Fields]) OR "feeding and eating disorders"[All Fields]) OR ("mood disorders"[MeSH Terms] OR ("mood"[All Fields] AND "disorders"[All Fields]) OR "mood disorders"[All Fields]) OR ("personality		

			disorders"[MeSH Terms] OR ("personality"[All Fields] AND "disorders"[All Fields]) OR "personality disorders"[All Fields]) OR ("schizophrenia spectrum and other psychotic disorders"[MeSH Terms] OR ("schizophrenia"[All Fields] AND "spectrum"[All Fields] AND "other"[All Fields] AND "psychotic"[All Fields] AND "disorders"[All Fields]) OR "schizophrenia spectrum and other psychotic disorders"[All Fields]) OR ("trauma and stressor related disorders"[MeSH Terms] OR ("trauma"[All Fields] AND "stressor"[All Fields] AND "related"[All Fields] AND "disorders"[All Fields]) OR "trauma and stressor related disorders"[All Fields])) OR ("severe mental illness"[All Fields] OR "serious mental illness"[All Fields] OR "SMI"[All Fields])		

Embase

Ovid Technologies, Inc. Email Service

Search for: 37 and 38 and 61

Results: 395

Database: Embase <1974 to 2024 April 26>

Search Strategy:

----

1 mortality/ or death/ (1160256)
2 survival/ (334597)
3 colon cancer/ or colon tumor/ or rectum carcinoma/ or colorectal cancer/ or colon carcinoma/ or rectum cancer/ or rectum tumor/ (370597)
4 mental disease/ (290264)
5 schizophrenia spectrum disorder/ (2470)
6 mood disorder/ (55382)
7 exp mood disorder/ (703392)
8 exp anxiety disorder/ (335322)
9 exp eating disorder/ (67360)
10 exp personality disorder/ (70308)
11 exp schizophrenia spectrum disorder/ (217424)
12 anxiety disorder/ (103239)
13 eating disorder/ (32798)
14 personality disorder/ (33009)
15 *personality disorder/ (12755)
16 schizophrenia.af. (244986)
17 bipolar disorder.af. (84058)
18 major depressive disorder.af. (46658)
19 anxiety.af. (532760)
20 eating disorder.af. (44397)
21 ptsd.af. (48727)
22 post-traumatic stress disorder.af. (23422)
23 borderline personality disorder.af. (10522)
24 (mortality or survival or death).af. (4796715)
25 (colorectal cancer or colon cancer or rectal cancer).af. (403179)
26 (severe and mental and illness).af. (19738)
27 (serious and mental and illness).af. (10660)
28 SMI.af. (14934)
29 26 or 27 or 28 (40416)
30 (schizophrenia or psychosis or bipolar disorder or major depressive disorder or anxiety or PTSD or eating disorders or personality disorder).af. (986284)
31 26 or 27 or 28 or 30 (1011660)
32 5 or 6 or 12 or 13 or 14 (202361)
33 1 or 2 (1403463)
34 3 and 29 and 33 (21)
35 3 and 30 and 33 (174)
36 3 and 32 and 33 (41)
37 1 or 2 or 24 (4796715)
38 3 or 25 (453376)
39 29 or 30 or 32 (1039450)
40 37 and 38 and 39 (1221)
41 3 and 33 and 39 (207)
42 3 and 4 and 33 (76)
43 "severe mental Illness".af. (7890)
44 "serious mental illness".af. (6046)
45 28 or 43 or 44 (25326)
46 7 or 8 or 9 or 10 or 11 (1131606)
47 30 or 46 (1441290)
48 3 and 33 and 47 (329)
49 45 and 48 (3)
50 30 or 32 or 45 (1032006)
51 37 and 38 and 50 (1209)
52 32 or 45 (226330)
53 37 and 38 and 52 (395)
54 30 and 37 and 38 (1020)
55 45 and 51 (163)
56 24 or 37 (4796715)
57 32 and 37 and 38 (236)
58 37 and 38 and 46 (1377)
59 30 and 37 and 38 (1020)
60 37 and 38 and 45 (163)
61 32 or 45 (226330)
62 37 and 38 and 61 (395)

****

Web of Science

	Search Query	Database	Results	Date Run
1	colorectal cancer (All Fields) OR colon cancer (All Fields) OR rectal cancer (All Fields)	Web of Science Core Collection	415849	Mon Apr 29 2024 16:01:47 GMT+0200 (Central European Summer Time)
2	mortality (All Fields) OR survival (All Fields) OR death (All Fields)	Web of Science Core Collection	3731270	Mon Apr 29 2024 16:02:19 GMT+0200 (Central European Summer Time)
3	"severe mental illness" (All Fields) OR "serious mental illness" (All Fields) OR SMI (All Fields)	Web of Science Core Collection	29273	Mon Apr 29 2024 16:02:58 GMT+0200 (Central European Summer Time)
4	#1 AND #2 AND #3	Web of Science Core Collection	125	Mon Apr 29 2024 16:04:04 GMT+0200 (Central European Summer Time)
5	mental illness (All Fields) OR mental disorder (All Fields)	Web of Science Core Collection	405085	Mon Apr 29 2024 16:07:10 GMT+0200 (Central European Summer Time)
6	schizophrenia (All Fields) OR psychosis (All Fields) OR bipolar disorder (All Fields) OR MAJOR DEPRESSIVE DISORDERS (All Fields) OR anxiety (All Fields) OR PTSD (All Fields) OR Personality disorders (All Fields) OR eating disorders (All Fields)	Web of Science Core Collection	902539	Mon Apr 29 2024 16:08:53 GMT+0200 (Central European Summer Time)
7	#6 OR #3	Web of Science Core Collection	922478	Mon Apr 29 2024 16:09:23 GMT+0200 (Central European Summer Time)
8	#7 AND #2 AND #1	Web of Science Core Collection	563	Mon Apr 29 2024 16:09:33 GMT+0200 (Central European Summer Time)
9	#5 OR #3 OR #6	Web of Science Core Collection	1116670	Mon Apr 29 2024 16:09:54 GMT+0200 (Central European Summer Time)
10	#9 AND #1 AND #2	Web of Science Core Collection	634	Mon Apr 29 2024 16:10:08 GMT+0200 (Central European Summer Time)

	Search Query	Database	Results	Date Run
11	#6 AND #1 AND #2	Web of Science Core Collection	453	Mon Apr 29 2024 16:10:30 GMT+0200 (Central European Summer Time)
12	#5 AND #2 AND #1	Web of Science Core Collection	143	Mon Apr 29 2024 16:10:40 GMT+0200 (Central European Summer Time)
13	#12 AND #4	Web of Science Core Collection	21	Mon Apr 29 2024 16:10:53 GMT+0200 (Central European Summer Time)
14	#12 OR #4	Web of Science Core Collection	247	Mon Apr 29 2024 16:11:20 GMT+0200 (Central European Summer Time)
15	#5 OR #3	Web of Science Core Collection	418021	Mon Apr 29 2024 16:11:57 GMT+0200 (Central European Summer Time)
16	#15 AND #1 AND #2	Web of Science Core Collection	247	Mon Apr 29 2024 16:12:07 GMT+0200 (Central European Summer Time)

CINAHL

S1	"severe mental illness or serious mental illness or severe and enduring mental illness"	Expanders - Apply equivalent subjects<br>Search modes - Boolean/Phrase	Interface - EBSCOhost Research Databases<br>Search Screen - Advanced Search<br>Database - CINAHL	0
S2	"severe mental illness or serious mental illness or severe and enduring mental illness"	Expanders - Apply equivalent subjects<br>Search modes - SmartText Searching	Interface - EBSCOhost Research Databases<br>Search Screen - Advanced Search<br>Database - CINAHL	1,701
S3	""severe mental illness" OR "serious mental illness" OR SMI"	Expanders - Apply equivalent subjects<br>Search modes - Boolean/Phrase	Interface - EBSCOhost Research Databases<br>Search Screen - Advanced Search<br>Database - CINAHL	3

S4	"mental illness"	Expanders - Apply equivalent subjects<br>Search modes - Boolean/Phrase	Interface - EBSCOhost Research Databases<br>Search Screen - Advanced Search<br>Database - CINAHL	63,367
S5	(MH "Mental Disorders")	Expanders - Apply equivalent subjects<br>Search modes - Boolean/Phrase	Interface - EBSCOhost Research Databases<br>Search Screen - Advanced Search<br>Database - CINAHL	67,710
S6	(MH "Mental Disorders") OR (MH "Adjustment Disorders") OR (MH "Gender Dysphoria") OR (MH "Intellectual Disability") OR (MH "Mental Disorders, Chronic") OR (MH "Mental Disorders Diagnosed in Childhood") OR (MH "Neurotic Disorders") OR (MH "Olfactory Reference Syndrome") OR (MH "Organic Mental Disorders") OR (MH "Paraphilic Disorders") OR (MH "Personality Disorders") OR (MH "Pregnancy Complications, Psychiatric") OR (MH "Psychophysiologic Disorders") OR (MH "Sexual Dysfunction, Psychological") OR (MH "Psychotic Disorders") OR (MH "Substance Use Disorders")	Expanders - Apply equivalent subjects<br>Search modes - Boolean/Phrase	Interface - EBSCOhost Research Databases<br>Search Screen - Advanced Search<br>Database - CINAHL	162,485
S7	(MH "Psychotic Disorders") OR (MH "Schizophrenia") OR (MH "Schizoaffective Disorder") OR (MH "Postpartum Psychosis") OR (MH "Paranoid Disorders") OR (MH "Organic Mental Disorders, Psychotic") OR (MH "ICU Psychosis") OR (MH "Capgras Syndrome") OR (MH "Affective Disorders, Psychotic") OR (MH "Passive-Aggressive Personality Disorder") OR (MH "Narcissistic Personality Disorder") OR (MH "Impulse Control Disorders") OR (MH "Histrionic	Expanders - Apply equivalent subjects<br>Search modes - Boolean/Phrase	Interface - EBSCOhost Research Databases<br>Search Screen - Advanced Search<br>Database - CINAHL	229,198

	Personality Disorder") OR (MH "Diogenes Syndrome") OR (MH "Dependent Personality Disorder") OR (MH "Borderline Personality Disorder") OR (MH "Compulsive Personality Disorder") OR (MH "Avoidant Personality Disorder") OR (MH "Antisocial Personality Disorder") OR (MH "Personality Disorders") OR (MH "Hypochondriasis") OR (MH "Body Integrity Identity Disorder") OR (MH "Body Dysmorphic Disorder") OR (MH "Somatoform Disorders") OR (MH "Munchausen Syndrome") OR (MH "Factitious Disorders") OR (MH "Multiple-Personality Disorder") OR (MH "Dissociative Disorders") OR (MH "Stress Disorders, Post-Traumatic") OR (MH "Social Anxiety Disorders") OR (MH "Phobic Disorders") OR (MH "Perinatal Mood and Anxiety Disorders") OR (MH "Panic Disorder") OR (MH "Obsessive-Compulsive Disorder") OR (MH "Generalized Anxiety Disorder") OR (MH "Anxiety Disorders") OR (MH "Seasonal Affective Disorder") OR (MH "Premenstrual Dysphoric Disorder") OR (MH "Depression") OR (MH "Affective Disorders") OR (MH "Neurotic Disorders")			
S8	(MH "Psychotic Disorders") OR (MH "Personality Disorders") OR (MH "Neurotic Disorders") OR (MH "Affective Disorders") OR (MH "Anxiety Disorders") OR (MH "Dissociative Disorders") OR (MH "Factitious Disorders") OR (MH "Somatoform Disorders") OR (MH "Schizophrenia") OR (MH "Affective Disorders, Psychotic")	Expanders - Apply equivalent subjects<br>Search modes - Boolean/Phrase	Interface - EBSCOhost Research Databases<br>Search Screen - Advanced Search<br>Database - CINAHL	70,463
S9	"colorectal cancer or colon cancer or bowel cancer or rectal cancer"	Expanders - Apply equivalent subjects<br>Search modes - Boolean/Phrase	Interface - EBSCOhost Research Databases<br>Search Screen - Advanced Search	0

			Database - CINAHL	
S10	"colorectal cancer or colon cancer or bowel cancer or rectal cancer"	Expanders - Apply equivalent subjects<br>Search modes - SmartText Searching	Interface - EBSCOhost Research Databases<br>Search Screen - Advanced Search<br>Database - CINAHL	11,838
S11	(MH "Colorectal Neoplasms") OR (MH "Neoplasia, Anal Intraepithelial") OR (MH "Anus Neoplasms") OR (MH "Rectal Neoplasms") OR (MH "Colorectal Neoplasms, Hereditary Nonpolyposis") OR (MH "Sigmoid Neoplasms") OR (MH "Colitis-Associated Neoplasms") OR (MH "Gardner Syndrome") OR (MH "Adenomatous Polyposis Coli") OR (MH "Colonic Neoplasms")	Expanders - Apply equivalent subjects<br>Search modes - Boolean/Phrase	Interface - EBSCOhost Research Databases<br>Search Screen - Advanced Search<br>Database - CINAHL	48,531
S12	"colorectal cancer OR colon cancer OR rectal cancer"	Expanders - Apply equivalent subjects<br>Search modes - Boolean/Phrase	Interface - EBSCOhost Research Databases<br>Search Screen - Advanced Search<br>Database - CINAHL	4
S13	(MH "Colorectal Neoplasms") OR (MH "Colonic Neoplasms") OR (MH "Rectal Neoplasms")	Expanders - Apply equivalent subjects<br>Search modes - Boolean/Phrase	Interface - EBSCOhost Research Databases<br>Search Screen - Advanced Search<br>Database - CINAHL	45,904
S14	"mortality OR survival OR death"	Expanders - Apply equivalent subjects<br>Search modes - Boolean/Phrase	Interface - EBSCOhost Research Databases<br>Search Screen - Advanced Search<br>Database - CINAHL	2

S15	"mortality or death or survival or outcome"	Expanders - Apply equivalent subjects<br>Search modes - Boolean/Phrase	Interface - EBSCOhost Research Databases<br>Search Screen - Advanced Search<br>Database - CINAHL	0
S16	"mortality or death or survival or outcome"	Expanders - Apply equivalent subjects<br>Search modes - SmartText Searching	Interface - EBSCOhost Research Databases<br>Search Screen - Advanced Search<br>Database - CINAHL	100,124
S17	"mortality or mortality rate or death or death rate"	Expanders - Apply equivalent subjects<br>Search modes - Boolean/Phrase	Interface - EBSCOhost Research Databases<br>Search Screen - Advanced Search<br>Database - CINAHL	0
S18	"mortality or mortality rate or death or death rate"	Expanders - Apply equivalent subjects<br>Search modes - SmartText Searching	Interface - EBSCOhost Research Databases<br>Search Screen - Advanced Search<br>Database - CINAHL	87,942
S19	"mortality rate or death rate or mortality or death"	Expanders - Apply equivalent subjects<br>Search modes - Boolean/Phrase	Interface - EBSCOhost Research Databases<br>Search Screen - Advanced Search<br>Database - CINAHL	0
S20	"mortality rate or death rate or mortality or death"	Expanders - Apply equivalent subjects<br>Search modes - SmartText Searching	Interface - EBSCOhost Research Databases<br>Search Screen - Advanced Search<br>Database - CINAHL	87,942
S21	(MH "Death") OR (MH "Survival") OR (MH "Mortality")	Expanders - Apply equivalent subjects<br>Search modes - Boolean/Phrase	Interface - EBSCOhost Research Databases<br>Search Screen - Advanced Search	107,011

			Database - CINAHL	
S22	(MH "Mortality") OR (MH "Cause of Death") OR (MH "Hospital Mortality") OR (MH "Survival Rate") OR (MH "Survival") OR (MH "Death") OR (MH "Treatment Outcomes") OR (MH "Fatal Outcome") OR (MH "Treatment Failure")	Expanders - Apply equivalent subjects<br>Search modes - Boolean/Phrase	Interface - EBSCOhost Research Databases<br>Search Screen - Advanced Search<br>Database - CINAHL	571,789
S23	S7 OR S8	Expanders - Apply equivalent subjects<br>Search modes - Boolean/Phrase	Interface - EBSCOhost Research Databases<br>Search Screen - Advanced Search<br>Database - CINAHL	229,198
S24	S13 AND S22 AND S23	Expanders - Apply equivalent subjects<br>Search modes - Boolean/Phrase	Interface - EBSCOhost Research Databases<br>Search Screen - Advanced Search<br>Database - CINAHL	41
S25	S14 OR S15 OR S16 OR S17 OR S18 OR S19 OR S20 OR S21 OR S22	Expanders - Apply equivalent subjects<br>Search modes - Boolean/Phrase	Interface - EBSCOhost Research Databases<br>Search Screen - Advanced Search<br>Database - CINAHL	571,835
S26	S9 OR S10 OR S11 OR S12 OR S13	Expanders - Apply equivalent subjects<br>Search modes - Boolean/Phrase	Interface - EBSCOhost Research Databases<br>Search Screen - Advanced Search<br>Database - CINAHL	48,596
S27	S1 OR S2 OR S3 OR S4 OR S5 OR S6 OR S7 OR S8	Expanders - Apply equivalent subjects<br>Search modes - Boolean/Phrase	Interface - EBSCOhost Research Databases<br>Search Screen - Advanced Search<br>Database - CINAHL	363,689

S28	S25 AND S26 AND S27	Expanders - Apply equivalent subjects<br>Search modes - Boolean/Phrase	Interface - EBSCOhost Research Databases<br>Search Screen - Advanced Search<br>Database - CINAHL	53

Supplementary file 5: Data extraction template

Identifying information

Review title or ID	
Study ID (surname of first author and year first full report of study was published e.g. Smith 2001)	
DOI	
Notes:	

General information

Date form completed (dd/	
Name/ID of person	
Reference citation	
Study author contact	
Publication type (e.g. full	
Supplementary materials	Unclear
Notes:	

Characteristics of included studies: Methods

	Descriptions as stated in report/paper		Location in text or source (pg & ¶/fig/table/ other)
Aim of study (e.g. efficacy, equivalence, pragmatic)			
Design (e.g. parallel, crossover, non-RCT)			
Duration of participation (from recruitment to last follow-up)			
Ethical approval needed/ obtained for study	Unclear		
Notes:			

Characteristics of included studies: Participants

	Description<br>Include comparative information for each intervention or comparison group if available		Location in text or source (pg & ¶/fig/table/ other)
Population description (from which study participants are drawn)			
Setting (including location and social context)			
Inclusion criteria			
Exclusion criteria			
Method of recruitment of participants (e.g. phone, mail, clinic patients)			
Comparator/Control			
Informed consent obtained	Unclear		
Cohort (or total pop. at start of study for NRCTs)			
SMI clusters (if applicable, no., type, no. people per cluster)			
Baseline imbalances			
Notes:			

Cancer type

	Description as stated in report/paper	Location in text or source (pg & ¶/fig/table/ other)
Exposure name		
Exposure definition (with diagnostic criteria if relevant)		
Notes:		

Outcomes

	Description as stated in report/paper	Location in text or source (pg & ¶/fig/table/ other)
Outcome name		
Outcome definition (with diagnostic criteria if relevant)		
Primary outcome		
Secondary outcome		
Notes:		

Data and analysis (other outcome)

	Description as stated in report/paper				Location in text or source (pg & ¶/fig/table/ other)
Comparison					
Outcome					
No. participant	SMI		Control		
Results:					
Any other results reported: survival since diagnosis by contact with mental health services	SMI result	SE (or other variance)	Control result	SE (or other variance)	
Statistical methods used and appropriateness of these					
Notes:					

Other information

	Description as stated in report/paper	Location in text or source (pg & ¶/fig/table/ other)
Key conclusions of study authors		
References to other relevant studies		
Correspondence required for further study information (from whom, what and when)		
Notes:		

Supplementary file 6: Excluded studies and grounds for exclusion

DOI	Covidence #	Study	Notes
10.1097/XCS.0000000000000954	#11	Katayama 2024	Exclusion reason: Wrong patient population;
10.1007/s00384-023-04358-0	#44	Orive 2023	Exclusion reason: Wrong patient population;
10.1007/s11845-023-03318-5	#45	Li 2023	Exclusion reason: Wrong patient population;
10.1016/j.jpsychores.2023.111162	#46	Avisar 2023	Exclusion reason: Wrong cancer type;
10.3390/ijerph192013548	#59	Soria-Utrilla 2022	Exclusion reason: Wrong patient population;
10.1007/s00520-022-07177-1	#73	Orive 2022	Exclusion reason: Wrong patient population;
10.1002/ags3.12421	#112	Kurashige 2021	Exclusion reason: Wrong comparator;
10.1097/PSY.0000000000000942	#114	Walker 2021	Exclusion reason: Wrong patient population;
10.1016/j.clinre.2020.07.016	#119	Zhou 2021	Exclusion reason: Wrong patient population;
10.3390/jcm9103174	#134	Trudel-Fitzgerald 2020	Exclusion reason: Wrong patient population;
10.1016/j.jpsychores.2020.110218	#136	Walker 2020	Exclusion reason: Wrong patient population;
10.1080/13548506.2019.1643032	#174	Xia 2020	Exclusion reason: Wrong patient population;
10.1002/da.22938	#178	Shoval 2019	Exclusion reason: Wrong patient population;
10.1097/COC.0000000000000529	#188	Lloyd 2019	Exclusion reason: Wrong patient population;
10.1111/ecc.12666	#231	Boursi 2018	Exclusion reason: Wrong comparator;
10.1136/bmj.j108	#233	Batty 2017	Exclusion reason: Wrong patient population;
10.1007/s11764-016-0553-4	#238	Kenzik 2016	Exclusion reason: Wrong outcomes;
10.1007/s11764-013-0286-6	#309	Mols 2013	Exclusion reason: Wrong patient population;
10.1177/070674371205700609	#325	Kisely 2012	Exclusion reason: Wrong patient population;
https://dx.doi.org/10.1053/j.gastro.2023.03.068	#483	Kilani 2023	Exclusion reason: No full text available;
https://dx.doi.org/10.1186/s12888-022-04191-9	#494	Benny 2022	Exclusion reason: Wrong outcomes;
https://dx.doi.org/10.1192/bjp.bp.117.198952	#687	Manderbacka 2017	Exclusion reason: Wrong cancer type;

https://dx.doi.org/10.1001/jamapsychiatry.2013.278	#749	Kisely 2013	Exclusion reason: Wrong patient population;
https://dx.doi.org/10.1097/01.PSYPHR.0000411896.67142.2d	#755	Moran 2012	Exclusion reason: Wrong study design;
https://dx.doi.org/10.1097/NCC.0b013e31819b59c0	#788	Tian 2009	Exclusion reason: Wrong patient population;
https://dx.doi.org/10.1007/s00268-007-9267-0	#796	Aoyanagi 2007	Exclusion reason: Wrong patient population;
10.1177/070674370805301107	#888	Kisely 2008	Exclusion reason: Wrong patient population;
10.1080/0284186X.2023.2282120	#901	Riedel 2023	Exclusion reason: Wrong patient population;
10.1111/jep.13318	#972	Sha 2020	Exclusion reason: Wrong patient population;
10.1097/NCC.0000000000001017	#1050	Tsai 2022	Exclusion reason: Wrong patient population;
10.1155/2013/269510	#1060	Sharma 2013	Exclusion reason: Wrong patient population;
10.1016/j.jss.2020.11.006	#1138	Oduyale 2021	Exclusion reason: Wrong cancer type;
10.1200/jco.2015.33.3_suppl.706	#1186	Cheung 2015	Exclusion reason: No full text available;
10.1002/pon.3886	#1330	ELCAPAStudyGrp 2016	Exclusion reason: Wrong patient population;
10.1080/0284186X.2018.1489144	#1474	Ørum 2018	Exclusion reason: Wrong patient population;
10.5388/aon.2015.15.1.37	#1484	Kim 2015	Exclusion reason: Wrong patient population;
10.1192/bjp.bp.114.156265	#1517	Ishikawa 2016	Exclusion reason: Wrong outcomes;
10.1136/bmjopen-2013-004295	#1523	Chang 2014	Exclusion reason: Wrong outcomes;
10.1245/s10434-015-4791-x	#1525	Wieghard 2015	Exclusion reason: Wrong outcomes;
10.1176/appi.ps.201900559	#1531	Ratcliff 2021	Exclusion reason: Wrong outcomes;
10.1177/0004867415577979	#1533	Kisely 2016	Exclusion reason: Wrong patient population;
10.2174/17450179-v17-e211208-2021-HT2-1910-8	#1536	Grassi 2023	Exclusion reason: Wrong study design;
10.1001/archpsyc.64.2.242	#1543	Osborn 2007	Exclusion reason: Wrong cancer type;
10.1002/cncr.28091	#1544	Musuuza 2013	Exclusion reason: Wrong patient population;
10.1016/j.socscimed.2009.07.040	#1546	Frederiksen 2009	Exclusion reason: Wrong patient population;

10.1002/pon.5853	#1554	Grassi 2021	Exclusion reason: Wrong study design;
10.1634/theoncologist.2019-0396	#1555	Lee 2020	Exclusion reason: Wrong patient population;
10.1016/j.schres.2015.11.010	#1559	Dickerson 2016	Exclusion reason: Wrong cancer type;
10.1016/j.ctro.2023.100618	#1560	Peters 2023	Exclusion reason: Wrong cancer type;
10.1016/j.jad.2014.08.025	#1561	Schoepf 2014	Exclusion reason: Wrong cancer type;
10.1186/1745-0179-4-23	#1562	Tidemalm 2008	Exclusion reason: Wrong cancer type;
10.1001/archpsyc.58.6.565	#1563	Druss 2001	Exclusion reason: Wrong cancer type;
10.1016/j.psychres.2013.07.042	#1564	Suokas 2013	Exclusion reason: Wrong cancer type;
10.1080/08039488.2020.1799431	#1567	Chen 2021	Exclusion reason: Wrong cancer type;
10.1192/bjp.187.4.334	#1572	Goldacre 2005	Exclusion reason: Wrong outcomes;
10.1177/0004867415577979	#1574	Kisely 2015	Exclusion reason: No full text available;
10.3390/healthcare10122366	#1575	Peritogiannis 2022	Exclusion reason: Wrong study design;
10.1111/j.1600-0447.1990.tb05466.x	#3008	Mortensen 1990	Exclusion reason: Wrong cancer type;
10.1192/bjp.163.2.183	#3010	Mortensen 1993	Exclusion reason: Wrong cancer type;
10.1111/j.1600-0447.1989.tb10255.x	#3013	Casadebaig 1989	Exclusion reason: Wrong cancer type;
10.1001/archpsyc.1985.01790240049005	#3015	Martin 1985	Exclusion reason: Wrong cancer type;
10.1192/bjp.136.3.239	#3016	Tsuang 1980	Exclusion reason: Wrong cancer type;
10.1192/bjp.130.2.162	#3017	Tsuang 1977	Exclusion reason: Wrong cancer type;
PMID: 24929569	#3018	Cunningham 2014	Exclusion reason: Wrong cancer type;
10.1016/j.psychres.2020.113702	#3021	Berardi 2021	Exclusion reason: Wrong cancer type;
10.1007/s00127-017-1468-8	#3024	Starace 2018	Exclusion reason: Wrong cancer type;
10.1097/NMD.0000000000000906	#3026	Berardi 2018	Exclusion reason: Wrong outcomes;
10.1093/schbul/sbt113	#3033	Dickerson 2014	Exclusion reason: Wrong cancer type;
10.1371/journal.pone.0055176	#3036	Nordentoft 2013	Exclusion reason: Wrong cancer type;
10.1097/SLA.0b013e31827b9b25	#3037	Liao 2013	Exclusion reason: Wrong cancer type;
10.1136/bmj.f2539	#3038	Lawrence 2013	Exclusion reason: Wrong patient population;
10.1186/1471-2458-13-834	#3040	Gissler 2013	Exclusion reason: Wrong cancer type;

10.1371/journal.pone.0024597	#3045	Laursen 2011	Exclusion reason: Wrong cancer type;
10.1136/bmj.d5422	#3046	Hoang 2011	Exclusion reason: Wrong cancer type;
10.1097/MLR.0b013e31820bf86e	#3047	Druss 2011	Exclusion reason: Wrong patient population;
10.1176/ps.2010.61.7.663	#3050	Piatt 2010	Exclusion reason: Wrong cancer type;
10.1136/jech.2008.083816	#3052	Hill 2010	Exclusion reason: Wrong patient population;
10.1186/1471-244X-10-77	#3053	Chang 2010	Exclusion reason: Wrong cancer type;
10.1192/bjp.bp.109.067512	#3055	Brown 2010	Exclusion reason: Wrong cancer type;
10.1016/S0140-6736(09)60742-X	#3057	Tiihonen 2009	Exclusion reason: Wrong cancer type;
10.1017/S0033291709005790	#3059	Grigoletti 2009	Exclusion reason: Wrong cancer type;
10.1038/sj.bjc.6604215	#3061	Frederiksen 2008	Exclusion reason: Wrong patient population;
10.1016/j.schres.2007.10.005	#3065	Capasso 2008	Exclusion reason: Wrong cancer type;
10.1186/1471-2407-7-20	#3066	Sloggett 2007	Exclusion reason: Wrong patient population;
10.1192/bjp.bp.106.025155	#3067	Ran 2007	Exclusion reason: Wrong cancer type;
10.1001/archpsyc.64.2.242	#3068	Osborn 2007	Exclusion reason: Wrong cancer type;
10.4088/jcp.v68n0612	#3069	Laursen 2007	Exclusion reason: Wrong cancer type;
10.1111/j.1467-842x.2006.tb00088.x	#3072	Shaw 2006	Exclusion reason: Wrong patient population;
10.1001/archpsyc.63.3.267	#3073	Daumit 2006	Exclusion reason: Wrong cancer type;
10.1007/s10552-004-7114-2	#3074	Menvielle 2005	Exclusion reason: Wrong patient population;
10.1192/bjp.187.6.552	#3076	Kisely 2005	Exclusion reason: Wrong cancer type;
10.1080/j.1440-1614.2005.01543.x	#3078	Drew 2005	Exclusion reason: Wrong cancer type;
10.1136/jech.57.4.301	#3079	Wrigley 2003	Exclusion reason: Wrong patient population;
10.1007/s00127-003-0635-2	#3080	Räsänen 2003	Exclusion reason: Wrong cancer type;
10.1016/s0920-9964(01)00308-5	#3081	Cohen 2002	Exclusion reason: Wrong cancer type;
10.1001/archpsyc.58.9.844	#3082	Osby 2001	Exclusion reason: Wrong cancer type;
10.1192/bjp.179.6.498	#3083	Joukamaa 2001	Exclusion reason: Wrong cancer type;
10.1192/bjp.179.5.438	#3084	Hansen 2001	Exclusion reason: Wrong cancer type;

10.1002/1097-0142(20010101)91:1+<208::aid-cncr6>3.0.co;2-e	#3086	Coleman 2001	Exclusion reason: Wrong patient population;
10.1016/s0920-9964(99)00191-7	#3087	Osby 2000	Exclusion reason: Wrong cancer type;
10.1007/s001270050248	#3088	Lawrence 2000	Exclusion reason: Wrong patient population;
10.1034/j.1600-0447.2000.101005382.x	#3089	Lawrence 2000	Exclusion reason: Wrong patient population;
10.1192/bjp.177.3.212	#3090	Brown 2000	Exclusion reason: Wrong cancer type;
10.1016/j.annepidem.2012.06.102	#3097	Wyman 2012	Exclusion reason: Wrong patient population;
10.3945/ajcn.115.127092	#3098	Walter 2016	Exclusion reason: Wrong patient population;
10.1007/s11605-019-04435-2	#3100	vandenBerg 2020	Exclusion reason: Wrong patient population;
10.1136/pgmj.2009.084566	#3102	Shack 2010	Exclusion reason: Wrong patient population;
10.1111/j.1572-0241.2003.07448.x	#3105	Rabeneck 2003	Exclusion reason: Wrong patient population;
10.1016/j.genhosppsych.2015.12.003	#3106	Pratt 2016	Exclusion reason: Wrong cancer type;
10.1016/S0140-6736(19)32316-5	#3108	Plana-Ripoll 2019	Exclusion reason: Wrong cancer type;
10.1016/j.genhosppsych.2006.05.006	#3111	Onitilo 2006	Exclusion reason: Wrong patient population;
10.1034/j.1600-0447.2000.00019.x	#3112	Muck-Jørgensen 2000	Exclusion reason: Wrong cancer type;
10.4088/JCP.13m08711	#3116	Kredentser 2014	Exclusion reason: Wrong cancer type;
10.1038/s41598-019-54677-y	#3117	Ko 2019	Exclusion reason: Wrong cancer type;
10.1521/pedi_2018_32_403	#3119	Kjær 2020	Exclusion reason: Wrong cancer type;
10.1007/DCR.0b013e3181974384	#3123	Iversen 2009	Exclusion reason: Wrong patient population;
10.1002/cncr.24270	#3124	Hébert 2009	Exclusion reason: Wrong patient population;
10.1192/bjp.bp.117.202606	#3125	Hayes 2017	Exclusion reason: Wrong cancer type;
10.1007/s00127-012-0612-8	#3127	Guan 2013	Exclusion reason: Wrong cancer type;
10.1016/S2215-0366(19)30126-9	#3132	Das-Munshi 2019	Exclusion reason: Wrong cancer type;
10.1016/j.schres.2018.10.020	#3137	Brink 2019	Exclusion reason: Wrong cancer type;
10.1038/bjc.2012.150	#3139	Batty 2012	Exclusion reason: Wrong cancer type;
10.1177/070674379103600401	#3142	Newman 1991	Exclusion reason: Wrong cancer type;

10.1111/j.1600-0447.1989.tb10254.x	#3147	Zilber 1989	Exclusion reason: Wrong cancer type;
10.1111/j.1600-0447.1985.tb02598.x	#3148	Wood 1985	Exclusion reason: Wrong cancer type;
10.1001/archpsyc.1980.01780220017001	#3150	Tsuang 1980	Exclusion reason: Wrong cancer type;
10.1002/gps.2833	#3156	Talaslahti 2012	Exclusion reason: Wrong cancer type;
10.1016/0010-440x(76)90045-6	#3160	Shinozaki 1976	Exclusion reason: Wrong cancer type;
10.1111/j.1600-0447.1979.tb04485.x	#3161	Saugstad 1979	Exclusion reason: Wrong cancer type;
10.1001/archpsyc.1985.01790240060006	#3171	Martin 1985	Exclusion reason: Wrong outcomes;
10.1111/j.1600-0447.1978.tb06904.x	#3183	Giel 1978	Exclusion reason: Wrong cancer type;
10.1017/s0033291704004118	#3185	Heilä 2005	Exclusion reason: Wrong cancer type;
PMID: 3964899	#3202	Black 1985	Exclusion reason: Wrong cancer type;
10.1176/ajp.126.4.470	#3205	Babigian 1969	Exclusion reason: Wrong cancer type;
10.1111/j.1600-0447.2006.00894.x	#3206	Amaddeo 2007	Exclusion reason: Wrong cancer type;
10.1001/archpsyc.1991.01810310017004	#3207	Allgulander 1991	Exclusion reason: Wrong cancer type;
10.1001/archpsyc.1986.01800070036005	#3208	Allebeck 1986	Exclusion reason: Wrong cancer type;

Supplementary file 7: Study duration, follow-up (min-max) and case fatality (%)

			SMI-Group				Control Group			
Study/Cohort type	Study period	Follow-up	N	CRC Cases	CRC Deaths	Case Fatality %	N	CRC Cases	CRC Deaths	Case Fatality %
**SMI Cohorts**										
Toender 2018	1978-2013	1-36 years	61,809	658	532	81	5,837,783	101,270	78,361	77
Launders 2022	2000-2018	0-18 years	69,632	376	60	16	278,528	1,841	228	12
Cheng 2023	2000-2019	0-19 years	107,481	540	268	50		2,818	930	33
**CRC Cohorts**										
Dalton 2008a, Dalton 2008b, Egeberg 2008	1994-2006	3-12 years		549	308	56		32,915	17,432	53
Liang 2020	1993-2018	10-15 years		358	81	23		2,038	518	25
Mahar 2020, Mahar 2021	2007-2013	3-9 years		740	377	51		23,767	9,827	41

Supplementary file 8: MR and MRR

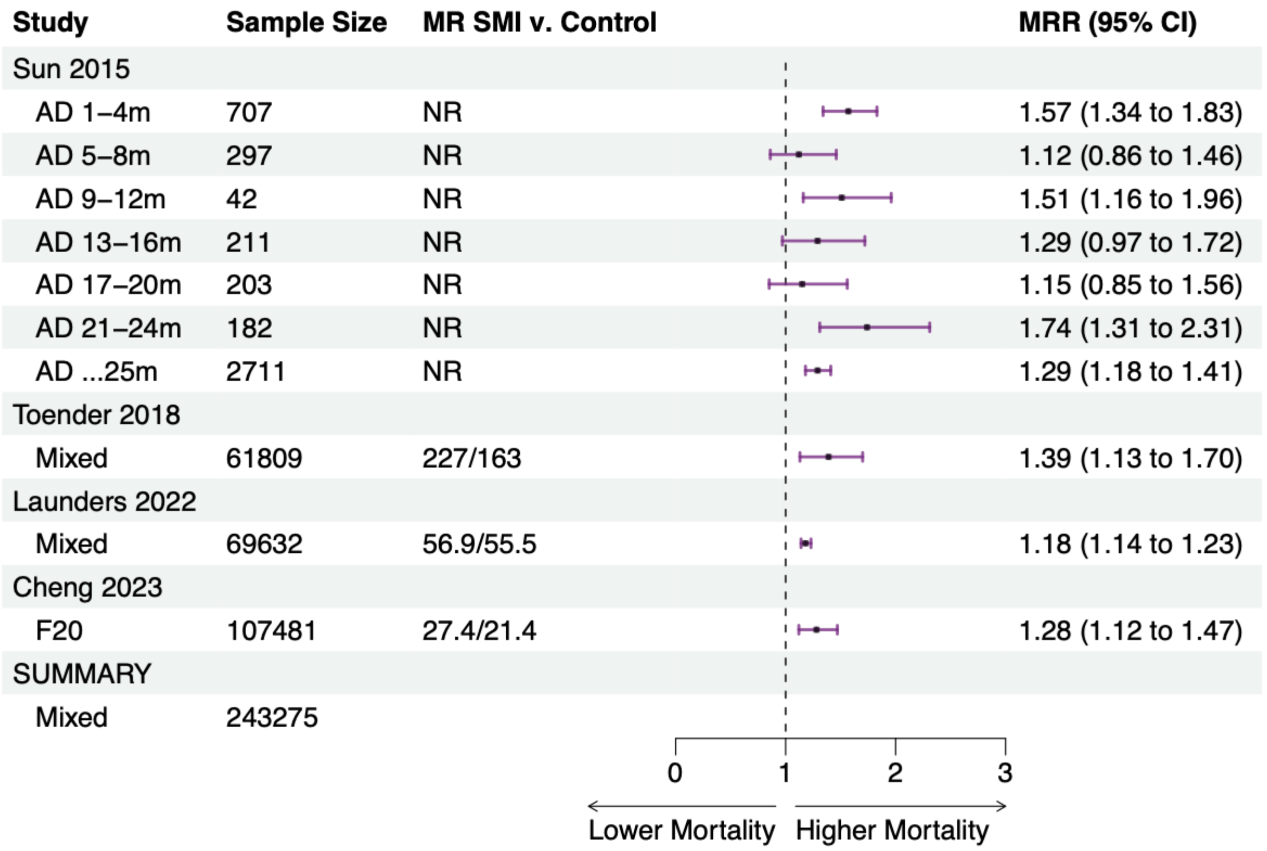

Figure 1. Forest plot showing extracted Mortality Rate Ratio with 95% Confidence Intervals for CRC-specific mortality. *Based the ICD-10: F20 refers to Schizophrenia, schizotypal and delusional disorders group (F20-F29); F30 refers to Mood [affective] disorders group (F30-39); F40 refers to Neurotic, stress-related and somatoform disorders group (F40-F48), F50 refers to Eating disorders; and F60 refers to Specific personality disorder. Mixed refers to a heterogenous group of SMI. The Sample size column displays the number of persons with SMI included in the study. The MR SMI v. Control column shows mortality rate (per 1,000PY in Toender 2018, per 1,000PY R in Lauders 2022, NR in Cheng 2023).*

Supplementary file 9: SMR and Excess Deaths

Study	Sample Size	Excess Deaths	SMR (95% CI)
Katz 1967			
Mixed	3365	–8.3	0.90 (0.72 to 1.12)
Saku 1995			
F20	4980	3.28	2.21 (0.89 to 4.59)
Tran 2009			
F20	3434	2.7	2.17 (0.80 to 4.82)
Perini 2014			
Mixed	100762	–0.43	0.98 (0.60 to 1.51)
Olfson 2015			
F20	1138853	279.59	1.70 (1.60 to 1.80)
Grassi 2021			
Mixed	12385	7.39	1.42 (0.92 to 2.09)
Chierzi 2023			
Mixed	101487	135.62	1.89 (1.68 to 2.13)
Drevinskaite 2024			
F20	8553	–6.29	0.81 (0.54 to 1.17)
SUMMARY			
Mixed	1373819	414.1	

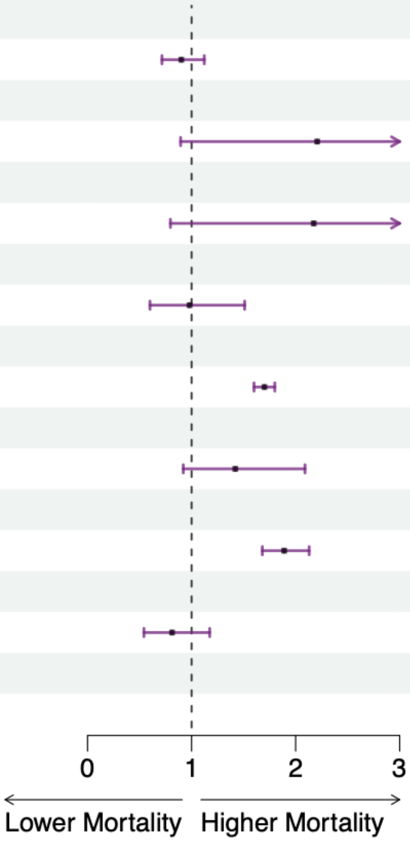

Figure 2. Forest plot showing extracted Standardized Mortality Ratio with 95% Confidence Intervals for CRC-specific mortality. *Based the ICD-10: F20 refers to Schizophrenia, schizotypal and delusional disorders group (F20-F29); F30 refers to Mood [affective] disorders group (F30-39); F40 refers to Neurotic, stress-related and somatoform disorders group (F40-F48), F50 refers to Eating disorders; and F60 refers to Specific personality disorder. Mixed refers to a heterogenous group of SMI. The Sample size column displays the number of PSMI included in the study. The Excess deaths column is calculated based on the extracted estimates for observed deaths in the SMI cohort and expected deaths in the general population using the formula excess deaths=obs.deaths – exp.deaths. The Summary SMR estimate is calculated using the cumulative observed and expected deaths from this cluster of studies and the formula for SMR=obs.deaths/exp.deaths with confidence intervals. Sample size refers to group with SMI.*

Supplementary file 10: HR

Study	Cohort type	Sample size	HR (95% CI)
Baillargeon 2011*			
Other SMI	Cancer	9322	1.03 (0.99 to 1.08)
F20	Cancer	3576	1.16 (1.10 to 1.24)
F30	Cancer	8261	1.04 (0.99 to 1.10)
Crump 2013a			
F30			
Men	SMI	2700	1.83 (0.92 to 3.67)
Women	SMI	3918	2.09 (1.21 to 3.60)
Crump 2013b			
F20			
Men	SMI	4787	1.87 (0.89 to 3.95)
Women	SMI	3490	2.35 (1.22 to 4.52)
Cunningham 2015*			
SMI	Cancer	33	1.89 (1.12 to 3.17)
AMI	Cancer	157	1.25 (0.89 to 1.75)
Klaassen 2019			
Outpatient	Cancer	50168	1.04 (1.02 to 1.07)
ER Visits	Cancer	1160	1.15 (1.01 to 1.31)
Inpatient	Cancer	641	1.71 (1.47 to 1.99)
Launders 2022			
Mixed	SMI	376	1.42 (0.84 to 2.39)
Liang 2020*			
F30	Cancer	358	0.90 (0.71 to 1.14)
Mahar 2020*			
Outpatient	Cancer	482	1.24 (1.04 to 1.48)
Inpatient	Cancer	258	1.69 (1.36 to 2.09)
Manderbacka 2018*			
F20	Cancer	751	
Men	Cancer	NR	1.72 (1.46 to 2.04)
Women	Cancer	NR	1.37 (1.20 to 1.57)
F30	Cancer	722	
Men	Cancer	NR	1.11 (0.91 to 1.36)
Women	Cancer	NR	1.10 (0.95 to 1.27)
Wootten 2024			
F20	SMI	5002	1.65 (1.35 to 2.01)
SUMMARY			
Mixed		96162	

All models are fully adjusted.

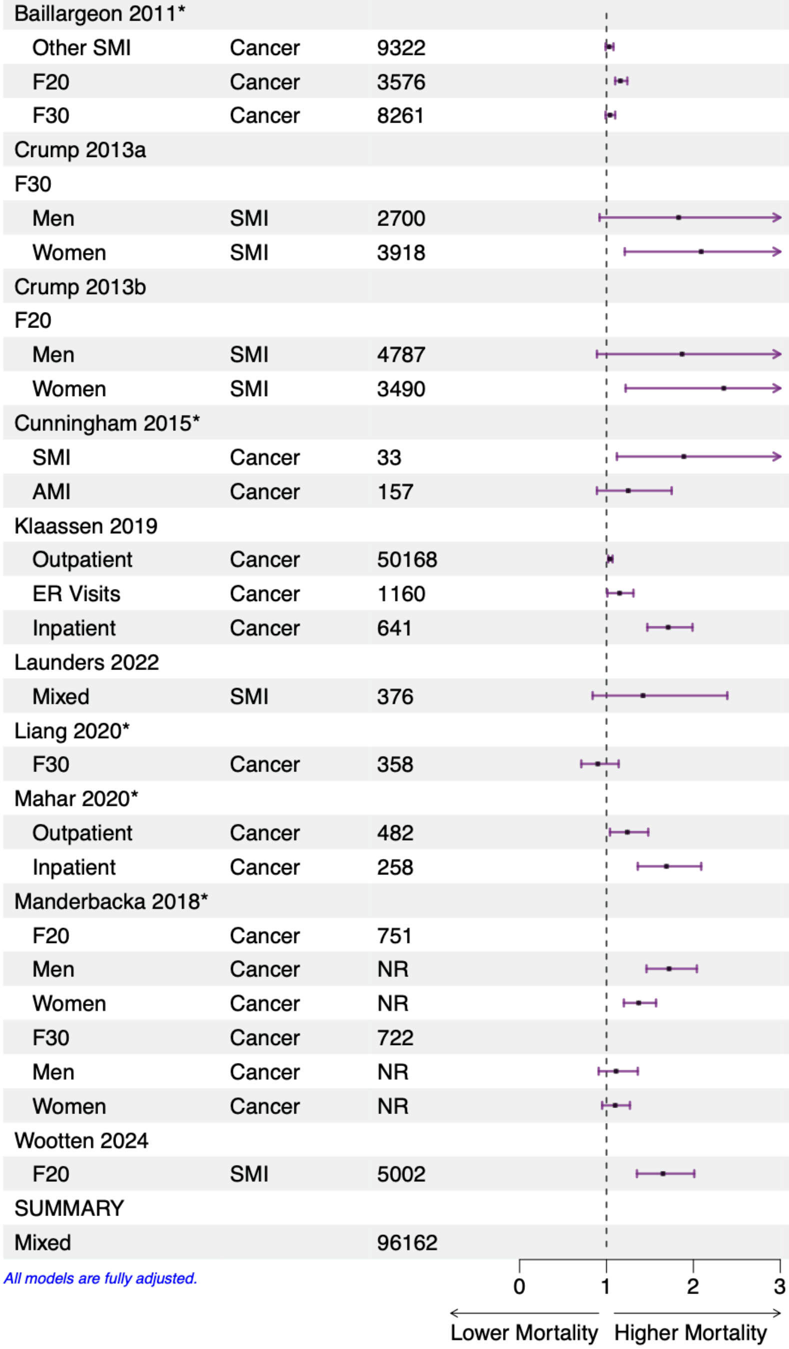

Figure 3. Forest plot showing extracted Hazard Ratio with 95% Confidence Intervals for CRC-specific mortality. *Based the ICD-10: F20 refers to Schizophrenia, schizotypal and delusional disorders group (F20-F29); F30 refers to Mood [affective] disorders group (F30-39); F40 refers to Neurotic, stress-related and somatoform disorders group (F40-F48), F50 refers to Eating disorders; and F60 refers to Specific personality disorder. Mixed refers to a heterogenous group of SMI. The Cohort type column displays the methodological approach, i.e., SMI: linking PSMI with later incident CRC and mortality; Cancer: linking persons with incident CRC to earlier SMI status; * indicates studies where CRC is the primary cancer. Sample size refers to group with SMI and CRC.*
